# Dynamic Phosphorylation of G9a Regulates its Repressive Activity on Chromatin Accessibility and Mitotic Progression

**DOI:** 10.1002/advs.202303224

**Published:** 2023-09-03

**Authors:** Qizhi Geng, Yue‐Yu Kong, Weizhe Li, Jianhao Zhang, Haoli Ma, Yuhang Zhang, Lin‐Tai Da, Yan Zhao, Hai‐Ning Du

**Affiliations:** ^1^ Hubei Key Laboratory of Cell Homeostasis College of Life Sciences Hubei Clinical Research Center of Emergency and Resuscitation Emergency Center of Zhongnan Hospital of Wuhan University Frontier Science Center for Immunology and Metabolism RNA Institute Wuhan University Wuhan 430072 China; ^2^ School of Life Sciences and Biotechnology Shanghai JiaoTong University Shanghai 200240 China; ^3^ Hubei Clinical Research Center of Emergency and Resuscitation Emergency Center of Zhongnan Hospital of Wuhan University Wuhan University Wuhan 430071 China; ^4^ Shanghai Center for Systems Biomedicine Shanghai JiaoTong University Shanghai 200240 China

**Keywords:** chromatin accessibility, G9a phosphorylation, mitotic progression, Plk1, PPP2CB

## Abstract

Phosphorylation of Ser10 of histone H3 (H3S10p), together with the adjacent methylation of Lys9 (H3K9me), has been proposed to function as a ‘phospho‐methyl switch’ to regulate mitotic chromatin architecture. Despite of immense understanding of the roles of H3S10 phosphorylation, how H3K9me2 are dynamically regulated during mitosis is poorly understood. Here, it is identified that Plk1 kinase phosphorylates the H3K9me1/2 methyltransferase G9a/EHMT2 at Thr1045 (pT1045) during early mitosis, which attenuates its catalytic activity toward H3K9me2. Cells bearing Thr1045 phosphomimic mutant of G9a (T1045E) show decreased H3K9me2 levels, increased chromatin accessibility, and delayed mitotic progression. By contrast, dephosphorylation of pT1045 during late mitosis by the protein phosphatase PPP2CB reactivates G9a activity and upregulates H3K9me2 levels, correlated with decreased levels of H3S10p. Therefore, the results provide a mechanistic explanation of the essential of a ‘phospho‐methyl switch’ and highlight the importance of Plk1 and PPP2CB‐mediated dynamic regulation of G9a activity in chromatin organization and mitotic progression.

## Graphical abstract

‘H3S10p‐H3K9me2 switch’ is important for mitotic chromatin architecture. This study uncovers that, during mitosis, the kinase Plk1 and the phosphatase PPP2CB dynamically phosphorylates/dephosphorylates the methyltransferase G9a to regulate H3K9me2 levels, chromatin accessibility, and mitotic progression, which highlights the importance of the crosstalk in chromatin organization and mitotic events.

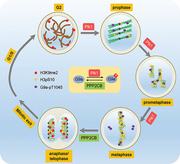

## 1 Introduction

Covalent modifications of histones play essential roles in regulating chromatin structure and genome integrity, such that epigenetic information can be faithfully inherited from parental cells into daughter cells. Among various modifications, histone H3K9 modifications have been illustrated as a determinant of heterochromatin formation and cell identity.^[^ ^1^ ^]^ Specifically, the trimethylation of H3K9 (H3K9me3) is found at constitutively pericentromeric heterochromatin and is viewed as a hallmark in transcriptionally silencing during cell fate determination and tissue identity.^[^ ^2^, ^3^ ^]^ By contrast, the dimethylation of H3K9 (H3K9me2) is positioned at the nuclear lamina‐associated heterochromatin and associates with gene silencing.^[^ ^4^, ^5^, ^6^ ^]^ Facilitated by a robust increase of H3S10p during prophase, the H3K9me2‐marked chromatin is released from the nuclear periphery into nucleoplasm, and reassembles at the nuclear periphery before mitotic exit.^[^ ^7^ ^]^ These findings underline the essential role of H3K9me2 as an architectural mitotic bookmarking.

H3S10 phosphorylation catalyzed by Aurora B kinase has been illustrated as a distinctive mark in mitotic progression, chromosome condensation, and segregation through regulating the interactions of chromatin with reader proteins.^[^ ^8^, ^9^, ^10^, ^11^, ^12^, ^13^, ^14^ ^]^ Its well‐recognized function involves in a ‘phospho‐methyl switch’, in which higher chromatin occupancy of H3S10p along the entire length of chromosomal arms at early mitosis hinders the binding of the heterochromatin protein HP1 to its adjacent H3K9me2/me3 marks.^[^ ^10^, ^11^ ^]^ The temporary dissociation of HP1‐bound peripheral heterochromatin from the nuclear lamina is essential for proper mitotic progression, as dysregulation of H3S10p by the introduction of non‐phosphorylable H3S10 alanine mutant leads to abnormal chromosome segregation and chromosome loss.^[^ ^15^, ^16^ ^]^ In addition, 30% of the genome consisting of early‐replicating domains is marked with H3S10p in interphase mouse embryonic stem cells, which is also anti‐correlated with H3K9me2‐marked heterochromatin regions.^[^ ^17^ ^]^ Interestingly, H3S10p and H3K9me2 are mutually exclusive, as disruption or inhibition of H3S10p results in ectopic spreading of H3K9me2 into adjacent euchromatic regions or recurrence of H3K9me2 in prometaphase chromosomes, whereas deletion of the H3K9 methyltransferases inversely leads to H3S10p‐associated chromatin domain expanding.^[^ ^17^, ^18^, ^19^ ^]^ These aforementioned observations reaffirm the importance of the strict regulation of ‘H3S10p‐H3K9me2 switch’. However, how H3S10p and H3K9me2 reciprocally antagonize is poorly understood.

Here, we find that Plk1 can regulate the catalytic activity of the methyltransferase G9a (also known as EHMT2) during early mitosis. Mitotic Plk1 protein phosphorylates G9a on T1045 residue to suppress G9a activity, resulting in decreased H3K9me2 levels, increased chromatin accessibility, and delayed mitotic progression from prometaphase toward the cytokinesis stage. Moreover, we show that phosphorylation of T1045 can be removed by the phosphatase PPP2CB subunit at late mitosis to reactivate G9a, thereby reestablishing H3K9me2‐marked peripheral heterochromatin and promoting mitotic exit. These results may provide a mechanistic explanation for the achievement of the ‘H3S10p‐H3K9me2 switch’ and highlight the importance of dynamic H3K9me2 regulation.

## 2 Results

### 2.1 Plk1 Kinase Phosphorylates G9a In Vitro and In Vivo

**Figure 1:**
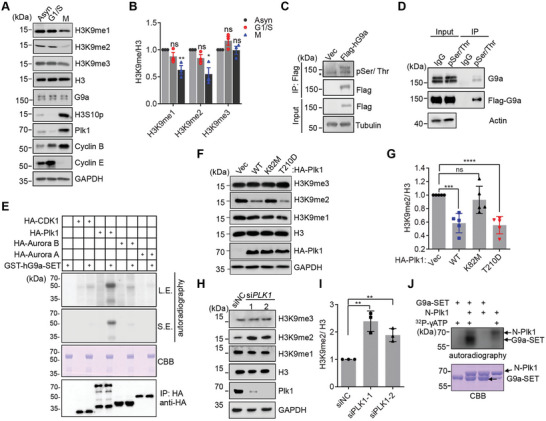
G9a can be phosphorylated by Plk1 both in vitro and in vivo. A,B) Western blots to examine H3K9me2 levels at asynchronous (Asyn), G1/S boundary, or mitosis (M) stage of HeLa S3 cells A), and different H3K9 methylation levels were quantified B). C) Western blots to examine the phosphorylation state of G9a in HeLa cells stably expressing Flag‐hG9a using an a‐pSer/Thr antibody. Asterisk represents non‐specific bands. D) Examination of the phosphorylation state of G9a by immunoprecipitation of phosphorylated proteins using an a‐pSer/Thr antibody. E) In vitro kinase assay examined which kinase can phosphorylate hG9a‐SET. The indicated kinases were enriched by immunoprecipitation from HEK293T cells expressing individual construct. Recombinant GST‐hG9a‐SET was detected by Commassie brilliant blue staining (CBB). Phosphorylation signals were examined by ^32^P‐labelled autoradiography. F,G) Western blots to examine H3K9me2 levels in HEK293T cells overexpressing various Plk1 constructs F), and relative H3K9me2 levels were quantified G). H,I) Western blots to examine various H3K9 methylation levels in HeLa S3 cells with knockdown of *PLK1* H), and relative H3K9me2 levels were quantified I). J) In vitro kinase assay to show that the N‐terminus of Plk1 containing the kinase domain (N‐Plk1) can phosphorylate G9a‐SET protein.

To validate that the ‘H3S10p‐H3K9me2 switch’ model does occur at mitosis, cell cycle‐dependent cellular distributions of various H3K9 methylation and H3S10p marks were examined. In concordance with previous results shown in HeLa, A549, and NIH3T3 cells,^[^ ^19^, ^20^ ^]^ immunofluorescent staining showed that H3K9me2 and H3S10p marks exclusively appeared in single HeLa S3 cell, in which H3K9me2 predominantly stained with interphase cells, whereas H3S10p only stained with mitotic cells (Figure S1A, Supporting Information). Meanwhile, we observed that H3K9me1 and H3K9me3 signals were co‐stained with H3S10p at each stage of the mitotic phase. In contrast, H3K9me2 signals were greatly decreased at prophase and metaphase and reappeared when cells entered anaphase and telophase (Figure S1B–D, Supporting Information). Consistently, when the cells that are arrested at different cell‐cycle stages were directly lysed and histones were extracted with SDS sample buffer, we repeatedly detected that H3S10p levels reached the highest at mitosis, and H3K9me1 and H3K9me2 levels inversely exhibited lower levels compared with that in G1/S phase by Western blots (**Figure** **1**A, B). The reduction of the H3K9me1/2 level at mitosis implies that the H3K9 methyltransferase activity might be modulated. It should note that the anti‐H3K9me2 antibody used in this work specifically recognized the H3K9me2 peptide, but not the dual H3K9me2S10p peptide. By contrast, an anti‐H3S10p antibody used recognized both H3S10p and the dual‐modified peptides, suggesting the possible existence of dual‐modified H3K9me2S10p histones in cells (Figure S1E, Supporting Information). However, the lack of commercially available anti‐H3K9me2S10p specifical antibody shields detailed investigation at the current stage.

The heterodimer comprised of G9a and G9a‐like protein (GLP) is the major enzyme for H3K9me1/2, and depletion or inhibition of either protein has been shown to result in a remarkable loss of H3K9me1/2.^[^ ^21^ ^]^ Thus, we next focused on exploring the regulation of G9a activity. Since multiple kinases become activated during the G2/M phase, we speculate that the G9a activity might be modulated through phosphorylation. Thus, Hela S3 cells stably expressing human Flag‐tagged G9a construct (Flag‐hG9a) were subjected to immunoprecipitation using either anti‐Flag antibody or an anti‐phospho Ser/Thr antibody as a bait, and the phosphorylation status of G9a was examined by immunoblotting. We noticed that both assays showed that G9a is phosphorylated in cells. (Figure 1C, D). To determine which kinases at the G2/M phase are responsible for G9a phosphorylation, in vitro kinase assay was conducted. Strikingly, we found that only Plk1, but not Cdk1, Aurora A, or Aurora B, was capable of phosphorylating recombinant hG9a segment containing the SET domain (hG9a‐SET), suggesting its kinase specificity (Figure 1E). Supporting the result, we have previously detected the interaction of G9a and Plk1 in cells.^[^ ^22^ ^]^ Strikingly, the phosphorylation levels of hG9a were negatively correlated with H3K9me2 levels, as expressing WT or the constitutively‐active T210D mutant of Plk1, but not catalytic‐inactive K82M mutant significantly reduced H3K9me2 levels (Figure 1F, G). Conversely, the knockdown of *PLK1* by two siRNA oligos remarkably elevated H3K9me2 levels in cells, suggesting a causal relationship between the methyltransferase activity of G9a and Plk1‐mediated phosphorylation event (Figure 1H, I). Furthermore, radioactive kinase assays indicated that active Plk1 protein containing the kinase domain (N‐Plk1) can directly phosphorylate recombinant GST‐G9a‐SET in vitro (Figure 1J). Altogether, we show that Plk1 can phosphorylate G9a in vitro and in vivo.

### 2.2 G9a is mainly Phosphorylated at Thr1045 Site

**Figure 2:**
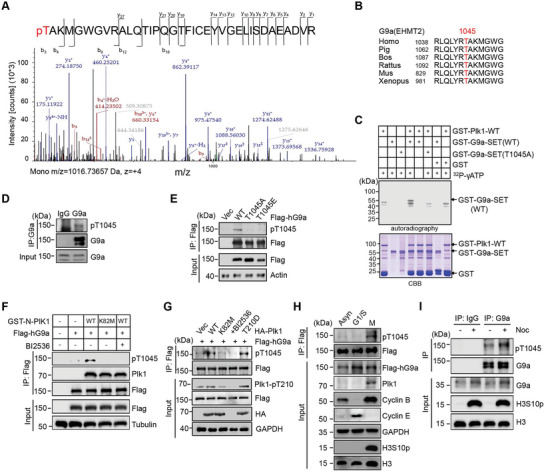
Identification of T1045 on G9a is phosphorylated by Plk1 during mitosis. A) Mass spectrum showed that G9a is phosphorylated at T1045. B) Sequence alignment of conserved T1045 sites of G9a in different species. C) In vitro kinase assay showed that T1045A mutation abolished Plk1‐mediated phosphorylation of G9a. D) T1045 phosphorylation of endogenous G9a was examined using an a‐pT1045 antibody. E) Western blots showed that only WT G9a, but not T1045A or T1045E mutant, was phosphorylated at T1045. F,G) Western blots showed that overexpression of WT Plk1, but not K82M mutant or addition of the Plk1 inhibitor BI2536, elevated phosphorylation levels of T1045 in vitro F) or in vivo G). H) Western blots showed that T1045 is phosphorylated at M phase in HeLa cells stably expressing Flag‐hG9a. I) Western blots showed that mitotic HEK293T cells by nocodazole treatment exhibited higher level of T1045 phosphorylation on endogenous G9a.

Next, we sought to identify the phosphorylation sites of G9a by Plk1. Given the large size of G9a protein (two major isoforms, observed molecular weight ≈130 kDa or 150 kDa, respectively) and hundreds of active kinases coexisted in cells, we decided to explore the potential phosphorylation sites of G9a using in vitro kinase assay. Therefore, recombinant GST‐G9a‐SET protein phosphorylated by Plk1 was subjected to mass spectrometry (MS) analysis, and phosphorylation on Thr1045 (pT1045) was identified (**Figure** **2**A). Sequence alignment indicated that this site is highly conserved across different species (Figure 2B). Interestingly, we noticed that T1045 of G9a is surrounded with the binding pocket of the methyl donor S‐adenosyl‐L‐methionine (SAM) based on resolved X‐ray crystal structure, implying a role of T1045 phosphorylation in regulating SAM recognition (Figure S2, Supporting Information). In vitro kinase assay corroborated that full‐length GST‐Plk1 protein effectively phosphorylates wild‐type G9a‐SET fragment, but not T1045A mutant (Figure 2C).

To validate whether T1045 phosphorylation does occur in cells, rabbit polyclonal anti‐pT1045 antibody was generated, and its specificity was carefully examined. Dot blot assay showed that the purified anti‐pT1045 antibody uniquely recognized a synthetic pT1045 peptide, but not the peptides containing T1045A and T1045E mutations, with a dose‐dependent manner (Figure S3A, Supporting Information). When the synthetic pT1045 peptide, instead of synthetic non‐phosphorylated T1045 peptide, was pre‐incubated with an anti‐pT1045 antibody, pT1045 signals were failed to be detected by Western blots (Figure S3B, Supporting Information). In cellular context, this antibody specifically recognized Flag‐G9a protein immunoprecipitated from cells (Figure S3C, Supporting Information). Using this antibody, we detected that endogenous G9a was phosphorylated at T1045 and mutating T1045 to other residues diminished the phosphorylated T1045 signals (Figure 2D,E).

### 2.3 T1045 Phosphorylation is Mediated by Plk1 mainly occurred at Mitosis

To further prove that pT1045 is catalyzed by Plk1, various recombinant GST‐N‐Plk1 fragments were individually incubated with Flag‐hG9a enriched from cells. As expected, only WT Plk1, but not the catalytic‐dead K82M mutant or addition of the Plk1 inhibitor BI2536, significantly enhanced pT1045 levels compared with the control (Figure 2F). Consistently, in vitro kinase assay showed that GST‐N‐Plk1 catalyzed T1045 phosphorylation of WT G9a‐SET, but barely on the indicated T1045 mutants (Figure S4A, Supporting Information). In HEK293T cells, overexpressing WT or constitutively active T210D form of Plk1 increased phosphorylation levels of T1045, whereas the catalytic inactive K82M mutant or addition of Plk1 inhibitor prevented T1045 phosphorylation (Figure 2G; Figure S4B, Supporting Information). Importantly, we found that pT1045 mainly occurs at mitosis, which was confirmed by experiments performed in cells transiently or stably expressing exogenous Flag‐hG9a construct, or in cells with endogenous G9a (Figure 2H,I; Figure S4C, Supporting Information). Collectively, these results indicate that pT1045 of G9a mainly occurs at M phase, at which stage that Plk1 is hyperactive.

### 2.4 T1045 Phosphorylation of G9a Decrease its Activity toward H3K9me2

**Figure 3:**
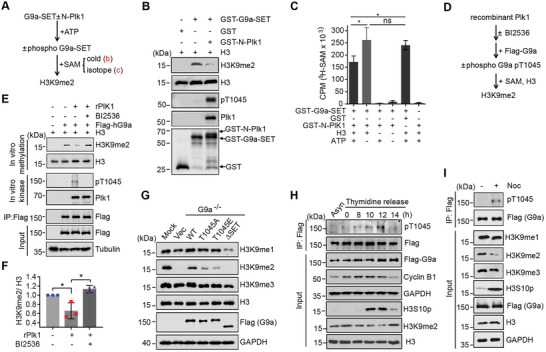
T1045 phosphorylation of G9a attenuated its catalytic ability on H3K9me2. A–C) Schematic diagram A) of sequential phosphorylation‐methylation assays performed by Western blots B) and by liquid scintillation counting C) using recombinant N‐Plk1 and G9a‐SET protein. Cold SAM: non‐radioactive; Isotope SAM: radioactive. CPM represents counts per minute. D–F) Schematic diagram D) of sequential in vitro phosphorylation‐methylation assays performed by Western blots E) using recombinant full‐length Plk1 protein and full‐length Flag‐G9a immunoprecipitated from HEK293T cells. Relative H3K9me2 levels were quantified F). G) Rescue of WT G9a, but not the indicated mutants of G9a, in HeLa S3 G9a‐/‐ cells showed a comparable H3K9me2 level relative to that in WT cells. H) HeLa S3 cells stably expressing Flag‐hG9a released from thymidine block were collected at the indicated time points, and pT1045 levels or H3K9me2 were examined by Western blots. I) Phosphorylation levels of T1045 or various H3K9 methylation states were examined by Western blots in HeLa S3 cells stably expressing Flag‐hG9a treated with or without nocodazole. Error bars denote the mean ± SD from three independent experiments. Unpaired *t*‐test, * P < 0.05, ns, not significant.

To explore whether pT1045 of G9a directly regulates its catalytic activity on H3K9me2, a series of in vitro reconstitution assays were conducted. First, we examined the catalytic activity of G9a with or without prior phosphorylation by Plk1 (**Figure** **3**A). We found that, after T1045 phosphorylation, H3K9me2 levels were obviously reduced examined by Western blots or radio‐labeled liquid scintillation counting (Figure 3B,C). Notably, without ATP, incubation of Plk1 with G9a remained high methylation levels on histone H3, implying the requirement of phosphorylation for inhibition of G9a activity (Figure 3C). Second, we examined whether inactive forms of N‐terminal fragment or full‐length Plk1, would influence G9a‐mediated H3K9 methylation (Figure 3D; Figure S5A, Supporting Information). In line with the results shown in Figure 3B, K82M mutant or addition of BI2536, compromised Plk1‐mediated inhibition of G9a, as increased H3K9me2 levels were observed in these assays (Figure 3E,F; Figure S5B, Supporting Information). Third, immunoprecipitated full‐length WT or various T1045 mutants of G9a was individually incubated with histone H3 in the presence of SAM donor. As predicted, the phosphomimic T1045E mutant of G9a displayed attenuated catalytic activity in terms of H3K9me2 level. Surprisingly, the phospho‐defective T1045A mutant also exhibited a reduced H3K9me2 level similar to T1045E, whereas T1045S mutant exhibits a comparable activity of H3K9me2 relative to that of WT G9a (Figure S5C,D, Supporting Information). We proposed that mutating Thr to Glu or Ala deforms local structures of binding pocket with SAM, but mutating Thr to Ser preserves a similar side chain structure.

To validate the hypothesis, a molecular dynamics (MD) simulation study was utilized, a method that computationally simulates the conformational changes introduced by phosphorylation of T1045 or various mutants of G9a (Figure S6A, Supporting Information). Based on MD simulation, relatively stable representative conformations of each molecule were computationally docked with a SAM molecule using their SAM binding pockets. Interestingly, we found that the pockets of WT and T1045S mutants retain enough space to occupy a SAM molecule, whereas the phosphorylation form of G9a, or T1045E and T1045A mutants fails to do so (Figure S6B, Supporting Information). Upon surveying the local structure of G9a, we noticed that a loop segment nearby the SAM binding pocket may interrupt SAM to fit into the binding hole of the G9a mutants, but this is only marginally affected by Thr‐to‐Ser substitution (Figure S6C, Supporting Information). Indeed, an average interaction energy between various forms of G9a and SAM calculated by molecular docking software showed that phosphorylation of T1045 markedly increased the interaction energy, but the T1045S mutant still sustained low energy compared to the WT form. We failed to obtain stabilized conformations of T1045E or T1045A mutant with SAM, suggesting that these mutants may severely disrupt the binding with SAM (Figure S6D, Supporting Information). All the molecular docking data are consistent with our experimental results in which phosphorylation of T1045 attenuates H3K9me2 level probably due to the disability of SAM binding. Consistently, we observed that, unlike WT G9a, expression of T1045 mutants in *G9a^−/‐^* cells could not rescue decreased H3K9me2 levels in vivo (Figure 3G). More importantly, pT1045 levels of G9a were cell‐cycle dependent, as we observed a peak occurred at mitosis, based on comparing pT1045 levels at different time points upon post‐release from thymidine or nocodazole block (Figure 3H, I). Therefore, we conclude that pT1045 of G9a compromises its methylation activity on H3K9me2.

### 2.5 T1045 Phosphorylation of G9a Attenuated its Activity of Gene Repression

**Figure 4:**
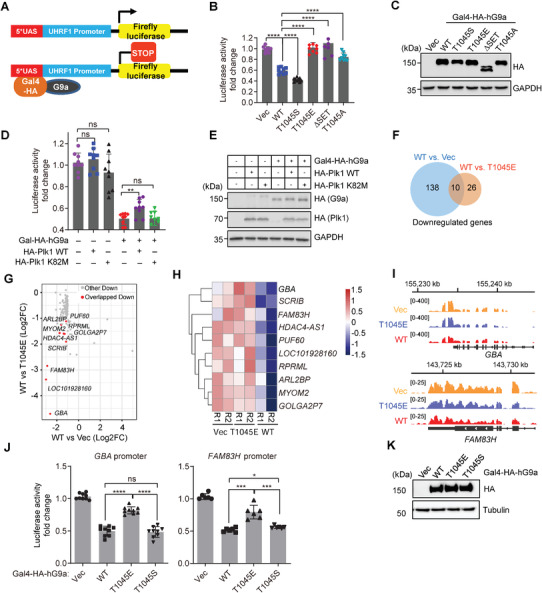
T1045 phosphorylation of G9a attenuated its repressive activity on gene repression. A) Schematic diagram of a reporter system to measure the effect of G9a on gene expression of luciferase (*LUC* gene) by targeting the *UHRF1* promoter. *GAL4*‐driven G9a construct specifically recognizes TATA boxes in the 5’‐*UAS* regions. B,C) Relative luciferase activities in cells expressing various G9a constructs were measured from three independent replicates with three technical repeats B), and protein levels of the indicated G9a constructs were examined by Western blots C). D,E) Relative luciferase activities in HEK293Tcells expressing the indicated Plk1 constructs were measured from three independent experiments each with three technical repeats D), and protein levels of the indicated cells were examined by Western blots E). F) Venn diagram showed the number of unique or shared genes downregulated in WT cells compared to cells expressing empty vector (Vec) or T1045E mutant, respectively [Log_2_(Fold change, FC) < −1, *P* < 0.05]. G) Scatter plots showed the downregulated genes in WT cells relative to HEK293T cells expressing Vec or T1045E mutant (*n* = 174). Red dots represent the overlapped genes between two groups. H) Heatmap depicted expression patterns of the ten overlapped genes in the indicated cells. Two independent replicates for each cell were presented. I) Genome browser tracks showed RNA‐seq signals at *FAM83H* and *GBA* gene loci in the indicated cells. J,K) Relative luciferase activities in HEK293T cells expressing the indicated G9a constructs were measured from multiply independent experiments J), and protein levels of the indicated cells were examined by Western blots K). Error bars denote the mean ± SD from three independent experiments. Unpaired *t*‐test, * *P* < 0.05, ** *P* < 0.01, *** *P* < 0.001, **** *P* < 0.0001, ns, not significant.

A negative correlation between H3K9me2 and gene expression was observed previously.^[^ ^5^ ^]^ To investigate whether pT1045 of G9a regulates its activity on gene expression, an established *UHRF1*‐luciferase reporter system was used.^[^ ^23^ ^]^ In this system, transcriptional levels of *UHRF1* regulated by various G9a constructs were examined (**Figure** **4**A). Consistent with previous studies,^[^ ^23^ ^]^ overexpression of WT G9a markedly repressed *UHRF1* promoter‐driven luciferase expression, whereas the SET domain‐deleted G9a mutant (∆SET) lost its repressive activity. As expected, T1045A and T1045E mutants displayed similar levels of luciferase activity as that of the control (Vec), whereas T1045S mutant showed more robust repressive activity than WT G9a (Figure 4B). Comparable protein levels expressed by different G9a mutants transfected in HEK293T cells were shown (Figure 4C). To verify whether Plk1 regulates the repressive activity of G9a, WT, or K82M Plk1 construct was co‐transfected with G9a in HEK293T cells, and the luciferase activities were monitored. We found that, overexpression of WT Plk1, but not K82M mutant, partially relieved the transcriptionally repressive activity of G9a (Figure 4D,E). Moreover, we found that the effects of various T1045 mutants on the luciferase activity were not influenced by Plk1 activity, implying the role of Plk1‐mediated pT1045 in regulating G9a activity (Figure S7A,B, Supporting Information).

To test the broad repressive effects of pT1045 on gene expression, RNA sequencing (RNA‐seq) analysis was performed. Compared to that in Vec‐overexpressed cells, transcriptional levels of 148 genes were significantly downregulated in WT G9a‐overexpressed cells. By contrast, 36 genes were downregulated in WT‐overexpressed cells compared to that in phosphomimic T1045E‐overexpressed cells (Figure S7C–E, Supporting Information). Among these downregulated genes in both groups, ten overlapped genes were presented (Figure 4F–H). Genome browser tracks showed that two of the representative genes were downregulated in WT‐overexpressed cells but not in T1045E‐overexpressed cells compared to control cells (Figure 4I). Luciferase reporter assays confirmed the candidate genes *GBA* and *FAM83H* were significantly repressed by WT G9a, but not T1045E mutant (Figure 4J,K). Intriguingly, three genes among the ten identified G9a‐repressed genes, including *SCRIB*, *ARL2*, and *MYOM2*, have been reported to link with mitotic progression, suggesting the role of G9a activity in mitosis.^[^ ^24^, ^25^, ^26^, ^27^ ^]^ Collectively, these results indicate that T1045 phosphorylation of G9a attenuates its repressive activity on gene expression.

### 2.6 T1045 Phosphorylation of G9a Decreases H3K9me2 Occupancy and Increases Chromatin Accessibility at Gene Promoters

**Figure 5:**
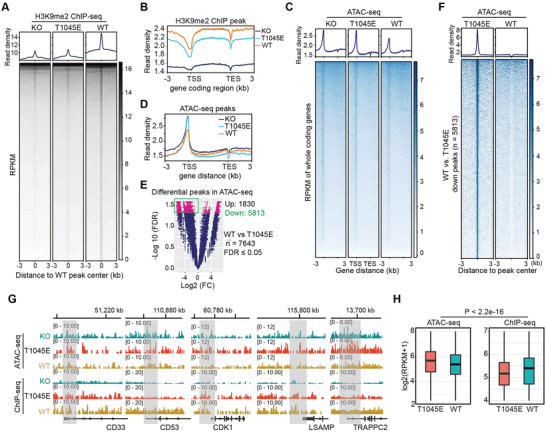
T1045 phosphorylation of G9a reduced H3K9me2 occupancy and increased chromatin accessibility at gene promoter regions. A) Averaged signal intensities (top) and heatmaps (bottom) showed the signals of H3K9me2 ± 3 kb from the center of peaks (*n* = 87,682) in the indicated cells. Each profile was plotted from two biological replicates. B) Profiles showed that averaged intensities of H3K9me2 ChIP‐seq in the gene coding regions ± 3 kb away from the normalized transcription start sites (TSS) and transcription end sites (TES) in the indicated cells. C) Averaged signal intensities (top) and heatmaps (bottom) showed the ATAC‐seq signals ± 3 kb away from TSS and TES in the indicated cells. Each profile was plotted from two biological replicates. D) Profiles showed that averaged intensities of ATAC‐seq in the gene coding regions ± 3 kb away from TSS and TES of the indicated cells. E) Volcano plots showed differential peaks from ATAC‐seq in *G9a* ^−/−^ cells stably expressing WT G9a relative to T1045E mutant. The downregulated ATAC‐seq peaks (*n* = 5,813) in WT sample were highlighted with green frame. F) Heatmaps showed ATAC‐seq differential signals from the center of ATAC‐seq peaks in T1045E mutant relative to that in WT cells. Each profile was plotted from two biological replicates. G) Genome browser tracks showed representative patterns of H3K9me2 ChIP‐seq and ATAC‐seq in 5 different genome loci of the indicated cells. H) Box plots compared the signals of ATAC‐seq and H3K9me2 peaks in WT and T1045E cells. *P*‐values were calculated by two‐sided *t*‐test.

To investigate the consequences of pT1045 of G9a in chromatin organization, we generated HeLa S3 cell lines stably expressing either WT or T1045E mutant under *G9a* knockout (KO) cells, and comparable protein levels of WT and T1045E were detected (Figure S8A, Supporting Information). H3K9me2 ChIP‐seq (chromatin immuno‐ precipitation combined with high‐throughput sequencing) was performed with two replicates for each cell line (Figure S8B, Supporting Information). The chromatin occupancy of H3K9me2 was significantly reduced in T1045E mutant compared with WT, but showed an equivalent enrichment compared to the KO control, suggesting that pT1045 of G9a may globally reduce H3K9me2 levels (**Figure** **5**A). Interestingly, we found that the majority population of H3K9me2 peaks occupied at intergenic (42 – 45%) and intron (≈43%) regions, but displayed ≈5% occupancy at adjacent gene promoters (< 2 kb) (Figure 5B; Figure S8C,D, Supporting Information). Given that less repressive epigenetic marks commonly reflected higher chromatin accessibility, we attempted to profile the effect of pT1045 on physical chromatin accessibility using ATAC‐seq (assay of transposase‐accessible chromatin with high‐throughput sequencing), in which two replicates for each cell line were used. We detected total 133 114 peaks in all three cell lines, and KO and T1045E cells exhibited slightly higher genome‐wide chromatin accessibility compared to WT (Figure S8E,F, Supporting Information). Notably, we observed that a large population of ATAC‐seq peaks were distributed at gene promoters (> 24%), where H3K9me2 marks were lost. By contrast, ≈30% percentage of ATAC‐seq peaks sustained at intergenic regions, where H3K9me2 was largely occupied, which probably arises from the substantial existence of intergenic regions rather than gene promoters (Figure S8G, Supporting Information). In line with largely occupied H3K9me2 peaks at intergenic regions, ATAC signals were displayed with a valley curve, suggestive of a closed chromatin state. We did not see obvious difference in chromatin accessibility between WT and T1045E at intergenic regions, in which modest reduction of H3K9me2 in T1045E cells might not be sufficient to influence heterochromatin structure at those regions (Figure S8H, Supporting Information). Conversely, with less H3K9me2 occupancy at the promoters, higher read density of ATAC signals was detected, indicative of opened chromatin conversion. More strikingly, KO and T1045E cells exhibited more opened chromatin state than that of WT cells, which strongly suggests pT1045 of G9a influences chromatin structure at gene promoters (Figure 5C,D).

Next, we focused on the role of T1045 phosphorylation in chromatin accessibility. We identified 7643 differential ATAC‐seq peaks, and 5813 peaks were downregulated in WT cells compared to T1045E cells (Figure 5E, F). Gene Ontology analysis of biological processes indicated that the 5813 downregulated peaks are associated with multiple cellular signaling events, such as cell growth and cell junction assembly, which may affect cell cycle progression (Figure S8I, Supporting Information). Genome browser tracks corroborated that the gene loci lacking chromatin accessibility were occupied with higher H3K9me2 marks in WT cells, but exhibited higher ATAC‐seq associated with lower H3K9me2 occupancy in T1045E and KO cells (Figure 5G). Moreover, the anti‐correlation of repressive H3K9me2 marks with open chromatin accessibility was detected in WT and T1045E mutants, further suggesting the roles of pT1045 of G9a in the reduction of H3K9me2 levels and openness of chromatin structures at gene promoters (Figure 5H).

### 2.7 T1045 Phosphorylation of G9a Regulates Mitotic Progression

**Figure 6:**
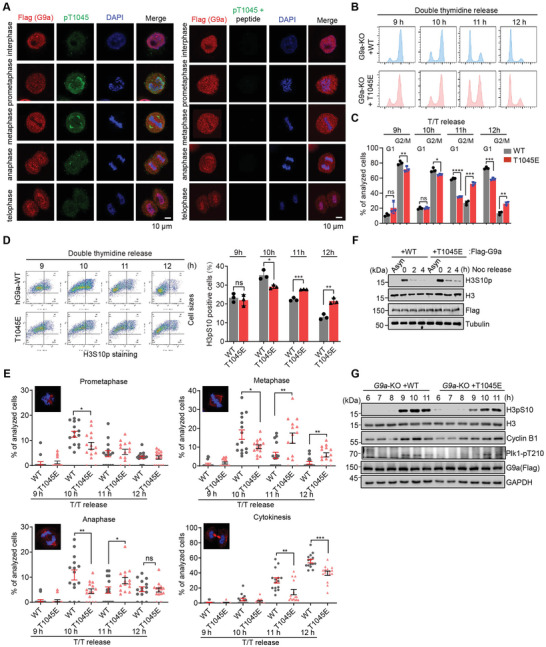
T1045 phosphomimics of G9a shows slower mitotic progression. A) Representative immunostaining images show the signals of pT1045 (green) and G9a (red) at different cell cycle stages in Flag‐G9a stably‐expressing cells (left panel). The specificity of pT1045 staining (green) is validated by pre‐incubation of pT1045 antibody with a phosphorylated T1045 peptide before immunostaining (right panel). DNA was stained with DAPI (blue). B,C) G9a^−/−^ cells expressing WT or T1045E mutant were synchronized by double‐thymidine (T/T) block and released to the indicated time points. Cell cycle profiles were analyzed using flow cytometry B), and quantification of cell populations at G1 phase or G2/M phase at different released time points were plotted from three biological replicates C). D) T/T released cells expressing WT or T1045E were stained with H3S10p. H3S10p positive cells at the indicated post‐released time points were analyzed by flow cytometry (left panel), and mitotic index was quantified. E) G9a^−/−^ cells expressing WT or T1045E mutant were synchronized by T/T block and released to the indicated time points. Cells were stained with alpha‐tubulin (red) and DAPI (blue) to determine different cell cycle stages. Five different images were captured at each time points from individual cells, and three independent experiments were performed (each dot represents an image). Cells in the indicated mitotic steps were manually counted. Error bars denote the mean ± SEM from three independent experiments. Representative immunostaining images are inserted. F) Western blots showed T1045E mutant delays dephosphorylation progression of H3S10p after post‐nocodazole release to the indicated time points. G) Western blots indicated that T1045E mutant exhibits a short delay of mitotic entry upon T/T release with the indicated time points. Error bars denote the mean ± SD from three biological experiments by unpaired *t*‐test. *P < 0.05, ** *P* < 0.01, *** *P* < 0.001, **** *P* < 0.001, ns, not significant.

Next, we aimed to determine the cellular function of T1045 phosphorylation of G9a. HeLa S3 cells stably expressing Flag‐tagged G9a were synchronized at G1/S boundary, then released with different time points, and interphase cells or mitotic cells through the consecutive phases (prometaphase, metaphase, anaphase, and cytokinesis) were stained with anti‐Flag and anti‐pT1045 antibodies. Unlike that G9a mainly occupied with chromatin throughout the whole cell cycle, we unexpectedly observed that pT1045 of G9a only appeared at early mitosis, especially at prometaphase and metaphase, and disappeared after anaphase (**Figure** **6**A, left panel). The signal specificity was validated by pre‐block of anti‐pT1045 antibody with pT1045 peptide before immunostaining assays (Figure 6A, right panel). Intriguingly, beside of staining with the chromatin, pT1045 signals seem to appear at centrosomes. To support that pT1045 generally occurs at early mitosis across different cell types, immunostaining was conducted. We observed that, in A549, HCT116, L02, and HepG2 cell lines, pT1045 of G9a were specifically shown with centrosomes at metaphase, but no signals were detected at interphase (Figure S9, Supporting Information), indicating the generality of G9a phosphorylation. Whether pT1045 functions in centrosomes needs to be investigated later. Nevertheless, immunostaining reinforces the potential role of this modification at mitosis.

To assess the involvement of pT1045 in different step of the cell cycle, G9a‐KO HeLa S3 cells stably expressing WT or T1045E mutant were synchronized with double thymidine and collected at the indicated time points upon post‐release, and then cells were stained with propidium iodide (PI) and subjected to flow cytometry analysis to determine cell populations. We observed that mitotic entry of majority of WT cells occurs at 9 – 10 h and exit from mitosis at 11 h post‐thymidine release. By contrast, T1045E mutant did not display an obvious difference at 10 h, but showed a remarkable delay of mitotic exit at 11 h post‐thymidine release, compared to the WT cells (Figure 6B,C). Furthermore, mitotic index by immunostaining cells with anti‐H3S10p antibody followed by flow cytometry analysis confirmed the delayed mitotic exit of T1045E mutant relative to WT cells (Figure 6D). These data are consistent with the appearance of pT1045 at early mitosis, but disappearance at late mitosis, suggesting the requirement of pT1045 dephosphorylation. Indeed, when synchronized cells were stained with an anti‐tubulin antibody and DAPI, the percentage of cells can be quantified according to their typical morphology (as shown in the representative images) at different times after post–thymidine release. As shown in Figure 6E, no significant difference between WT and T1045E cells was observed at the prometaphase. However, 10 h after release, the number of WT cells in the metaphase and the anaphase was higher than that of T1045E cells. Moreover, 11 – 12 h after release, the percentage of WT cells in cytokinesis was much higher than that of T1045E cells, which indicates that the persistent phosphomimic T1045E mutant cells markedly delayed late mitosis progression (Figure 6E). Supporting this point, synchronized cells at prometaphase exhibited different H3S10p patterns after nocodazole release in WT and T1045E cells, indicating a delayed mitotic exit in T1045E mutant (Figure 6F). Mitotic entry patterns were also compared between WT and T1045E, in which a short delay in T1045E mutant was detected by visualizing the signal peaks of H3S10p and pT210 of Plk1 (Figure 6G). Altogether, these results suggest that dynamic T1045 phosphorylation of G9a regulates mitotic progression.

### 2.8 PPP2CB Dephosphorylates pT1045 of G9a to Regulate its Catalytic Activity on H3K9me2

**Figure 7:**
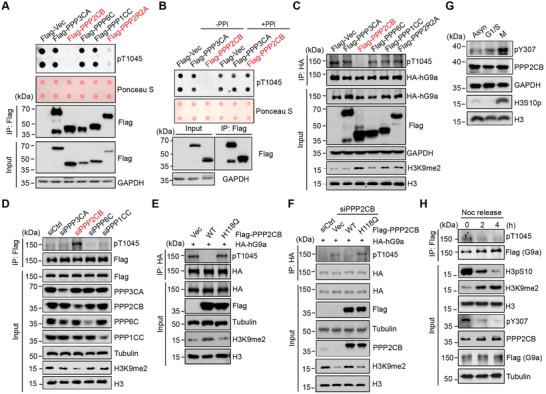
PPP2CB phosphatase is required for dephosphorylation of G9a on T1045 during late mitosis. A) Dot blot assay showed that both the regulatory and catalytic subunits of PPP2CB from cells dephosphorylate pT1045 in vitro. B) Dot blot assay showed that inhibition of PPP2 activity by adding the phosphatase inhibitors blocked dephosphorylation of pT1045 in vitro. C) Western blots showed that overexpression of PPP2CB, but not other phosphatases, dephosphorylates pT1045 of G9a in HEK293T cells. D) Western blots showed that siRNA knockdown of *PPP2CB* increased pT1045 levels of G9a in HEK293T cells. E) Western blots showed that overexpression of WT, but not catalytic‐inactive of PPP2CB, dephosphorylates pT1045 of G9a. F) Western blots showed that expressing WT PPP2CB, but not catalytic‐dead H118Q mutant in cells in *PPP2CB* knockdown cells restored elevated pT1045 levels. G) PPP2CB phosphatase activity was examined at different cell cycle stages by immunoblotting with an a‐pY307 PPP2CB antibody. H) PPP2CB and G9a activities were examined in HeLa S3 cells with nocodazole arrest and release for the indicated time points by immunoblotting against anti‐a‐pY307 and anti‐pT1045 and anti‐H3K9me2 antibodies.

To ensure proper mitotic exit, how pT1045 of G9a is dynamically modulated at late mitosis was further investigated. To do this, a proximity labeling approach was applied to identify the interactors of G9a by genetic fusion of G9a with biotin ligase BirA (termed as Flag‐BirA‐G9a) in living cells.^[^ ^28^ ^]^ By supplemented with biotin, cell extracts were subjected to immunoprecipitation using streptavidin beads, and mass spectrometry analysis was performed (Figure S10A, Supporting Information). Among hundreds of identified G9a‐interacting proteins, five subunits from four different phosphatases were identified, including the beta‐isoform of the catalytic subunit of PPP2 (PPP2CB), the gamma isoform of the catalytic subunit of PPP1 (PPP1CC), the B55alpha regulatory subunit of PPP2 (PPP2R2A), the alpha isoform of the catalytic subunit of PPP3 (PPP3CA), and the catalytic subunit of PPP6 (PPP6C) (Figure S10B, Supporting Information). To examine whether they could dephosphorylate pT1045, Flag‐tagged constructs encoding aforementioned phosphatases were expressed in cells, and immunoprecipitated proteins were individually incubated with synthetic pT1045 peptide. Dot blot assay showed that only two PPP2CB subunits were capable to eliminate the phosphorylation signal of T1045 (**Figure** **7**A). Diminishment of pT1045 signals was specific, as adding a cocktail of phosphatase inhibitors in the in vitro phosphatase reactions blocked the reduction (Figure 7B). Interestingly, overexpression of PPP2CB, but not other subunits of phosphatases, markedly reduced pT1045 level, accompanied by increased H3K9me2 level (Figure 7C). Intriguingly, overexpression of PPP2R2A did not reduce pT1045 level as predicted, which might arise from lacking sufficient catalytic PPP2CB subunit in the cell. Consistently, we found that knockdown of *PPP2CB* by siRNA transfection obviously increased pT1045 level and consequently decreased H3K9me2 level, compared to the control (Figure 7D). To rule out that co‐contaminant proteins or side effects from manipulation of PPP2CB might influence the outcome, WT or catalytic‐inactive H118Q mutant of PPP2CB was expressed in cells. As expected, only active WT construct efficiently dephosphorylated pT1045 signal (Figure 7E). Rescue experiment by adding back siRNA‐resistant WT PPP2CB construct, but not H118Q mutant, in si*PPP2CB* cells restored H3K9me2 level as similar as that in control cells (Figure 7F). The data together indicate that PPP2CB is required for dephosphorylation of pT1045.

Last, we wanted to examine the effect of PPP2CB on G9a during mitosis. Thus, the catalytic activities of PPP2CB from HeLa S3 cells at the indicated cell‐cycle stages were examined by Western blot using an anti‐phosphorylated Tyr‐307 (pY307) antibody. Consistent with previous studies,^[^ ^29^ ^]^ we found that PPP2CB was phosphorylated at Y307 coincident with hyperphosphorylation of H3S10, suggesting that it was inactive at metaphase (Figure 7G). When mitotic cells were released from nocodazole arrest, we observed that PPP2CB activity was gradually increased, detected by reduced levels of pY307, accompanied by decreased levels of pT1045 and elevated levels of H3K9me2, suggesting that PPP2CB counteracts with Plk1 to regulate the G9a activity (Figure 7H). To examine whether the effect of PPP2CB on G9a is cell‐type specific, four aforementioned cell lines were treated with the specific PPP2CB inhibitor LB100, and the pT1045 signals were monitored by immunostaining. Consistently, cells sustained strong pT1045 signals at late mitosis rather than in non‐treated cells, suggesting dephosphorylation of pT1045 by PPP2CB does occur at this stage (Figure S10C, Supporting Information).

## 3 Discussion

Numerous studies have reported that mitotic‐associated phosphorylation of H3S10 antagonizes H3K9me2 marks across different organisms,^[^ ^5^, ^7^, ^17^, ^19^, ^20^, ^30^, ^31^ ^]^ but the regulatory mechanism of ‘H3S10p‐H3K9me2’ switch has been unexplored. In this work, we demonstrate that activated Plk1 kinase phosphorylates T1045 of G9a, which is located at the binding pocket of SAM donor during early mitosis. This event temporarily inhibits G9a methylation activity and leads to reduction of H3K9me2 levels and openness of chromatin accessibility. Subsequently, we find that phosphorylated G9a can be removed by PPP2CB during late mitosis that reactivates G9a activity and ensures appropriate mitotic exit.

H3K9me2 and H3S10p marks are co‐regulated during cell cycle progression. During prophase, elevated H3S10p temporarily shields H3K9me2 modification, thereby facilitating the dissociation of the H3K9me2‐marked peripheral heterochromatin and the rearrangement of the genome inside the nucleus to form H3S10p‐marked chromosome arms, which is pivotal for mitotic progression and chromosome segregation.^[^ ^8^, ^15^, ^32^ ^]^ In support of the antagonistic effect, several laboratories have reported that H3S10p‐bound chromatin is mutually excluded with H3K9me2‐marked chromatin examined by Western blotting, immunostaining and genome‐wide profiling.^[^ ^5^, ^17^, ^19^, ^20^ ^]^ Different from these observations, Poleshko et al. indicate that H3K9me2 persists through mitosis and co‐exists with H3S10p at mitotic chromatin in murine C2C12 cells.^[^ ^7^ ^]^ We speculate that different cell types might have different mitotic phenotypes. Nevertheless, we herein find that G9a activity, instead of G9a protein levels, is attenuated via Plk1‐mediated phosphorylation at early mitosis.

As a master mitotic kinase regulating multiple aspects of the cell division process, Plk1 functions in mitotic entry, chromosome segregation, spindle assembly checkpoint, and cytokinesis by phosphorylation of various substrates that localize at certain cellular regions, including centrosomes, kinetochore, spindle poles, and midbody.^[^ ^33^, ^34^, ^35^, ^36^ ^]^ Concomitantly, previous studies showed that, upon mitotic entry, Plk1 localizes to the nuclear envelope and acts with Cdk1 to phosphorylate the central nucleoporins, inducing mitotic nuclear pore complex disassembly during prophase.^[^ ^37^, ^38^ ^]^ Given that G9a is also enriched peripheral heterochromatin at the nuclear lamina, it is very likely that Plk1 acts at G9a to de‐attach heterochromatin from the nuclear lamina into the nucleus.^[^ ^7^ ^]^ It is worth to note that pT1045 immunostaining signals were detected with chromatin and centrosomes during prometaphase and metaphase, at which stages Plk1 signals are also appeared with centrosomes, further suggesting the regulatory relation of Plk1 and phosphorylation of G9a.^[^ ^34^ ^]^ Thus, Plk1‐mediated G9a phosphorylation is pivotal for achievement of ‘H3S10p‐H3K9me2’ switch at early mitosis. Interestingly, G9a phosphorylation is also occurred upon DNA damage stress, in which casein kinase 2 phosphorylates G9a at another site to facilitate G9a chromatin association,^[^ ^39^ ^]^ indicating that dynamic phosphorylation events on G9a by different kinases modulate divergent functions. It should be noted that the reduction of H3K9me2 levels at early mitosis may be also mediated by the activation of the demethylases. It would be of interest to test this hypothesis later.

Substantial evidence has illustrated that G9a‐mediated H3K9me2 is biologically required for maintaining DNA methylation, high‐order chromatin structure, and transcriptional silencing.^[^ ^1^, ^40^, ^41^ ^]^ Therefore, balancing proper G9a methylation activity under different circumstances is vitally necessary. In this study, we found that the catalytic activity of G9a is regulated through dynamic phosphorylation of T1045. Several lines of evidence support our conclusion. First, both mass spectrometry analysis and the specific antibody recognizing pT1045 corroborate the existence of T1045 phosphorylation. Second, using a group‐based prediction system GPS 6.0 (www.gps.biocuckoo.cn), Plk1‐mediated T1045 phosphorylation is on the potential lists.^[^ ^42^ ^]^ Third, altering Plk1 kinase activity modulates pT1045 levels and G9a‐mediated H3K9me2 levels in vitro and in vivo. Fourth, phosphomimic T1045E mutant reduces genome‐wide H3K9me2 levels particularly at mitosis. Last, the removal of T1045 phosphorylation by PPP2CB restores higher H3K9me2 level. Of note, although molecular docking by computer modeling suggests that the phosphorylation of T1045 hinders the association of G9a with SAM, the precise structure of G9a bearing phosphorylated T1045 is essential for explanation of the detailed mechanism.

It is thought that, during mitosis, chromatin condenses, and transcription is generally silenced to facilitate chromosome segregation, however, genome accessibility is widely preserved, especially at the proximal promoters.^[^ ^43^, ^44^ ^]^ We herein found that decreased H3K9me2 coincides with more opened chromatin accessibility at 2521 gene loci, suggesting that transcription of certain mitotic genes is activated at the beginning of mitosis, probably for mitotic progression. Actually, several studies have demonstrated that hundreds of genes, besides centromere genes, exhibited >twofold transcriptionally activated in mitosis than in asynchronous cells,^[^ ^45^, ^46^, ^47^ ^]^ which connects opened chromatin accessibility with transcription activation that may be required for appropriate mitotic progression. Certainly, the majority of mitotic chromosomes are highly compacted and transcriptional repression throughout mitosis, until a specific gene expression program is reactivated upon mitotic exit.^[^ ^46^, ^47^ ^]^ Whether transient inactivation of G9a by Plk1 phosphorylation is directly linked with mitotic gene activation should be carefully inspected.

Dephosphorylation of H3S10 at mitotic exit facilitates the reestablishment of H3K9me2‐marked chromatin architecture, as it has been shown that constitutive expression of the phosphomimic H3S10E mutant leads to nuclear distribution of heterochromatin, but not at the nuclear periphery.^[^ ^7^ ^]^ The dephosphorylation of H3S10 in vivo requires the phosphatase PP1.^[^ ^48^ ^]^ Here we identify the phosphatase PPP2CB is required for dephosphorylation of G9a on T1045 and reactivation of G9a to reform H3K9me2 marks. PPP2CB is a catalytic subunit of the protein phosphatase 2A (PP2A), which belongs to protein serine/threonine phosphatase family and is conserved across eukaryotic evolution.^[^ ^49^ ^]^ PP2A is a master regulator of the cell cycle, and a broad spectrum of proteins have been reported as PP2A substrates that are involved in different cell cycle stages.^[^ ^50^, ^51^ ^]^ However, inhibition of PP2A is required for mitotic entry by diminishing its antagonistic effect on Cdk1.^[^ ^50^ ^]^ By contrast, PP2A functions in nuclear envelope reassembly and regulates spindle checkpoint and chromosome segregation,^[^ ^52^, ^53^ ^]^ which suggests that appropriate activation of PP2A at late mitosis is essential for mitotic exit. Our finding regarding G9a as the substrate of PPP2CB supplements a novel role of PP2A in heterochromatin reassembly.

In conclusion, our study reveals a dynamic regulatory mechanism of G9a activity during M phase, which contributes to altering H3K9me2 levels and heterochromatin reorganization. Since upregulated G9a has been observed in different type of cancer and is closely linked with poor prognosis,^[^ ^54^ ^]^ this study may provide a new cancer therapeutics by targeting T1045 phosphorylation of G9a. At cellular level, the ‘phospho‐methyl switch’ may represent a widespread mechanism to regulate chromatin organization, cell cycle progression, and epigenetic inheritance. Elucidation of the regulatory crosstalk between the two adjacent histone modifications may reveal unrecognized epigenetic mechanisms modulating the division of cancer cells and stem cells.

## 4 Experimental Section

### Cell Culture, Transfection, and Treatment

HEK293T, HeLa, HeLa S3, A549, HCT116, L02, and HepG2 cell lines were cultured in DMEM (Dulbecco's modified Eagle's medium), supplemented with 10% fetal bovine serum (Cat. A0500‐3011, Cegrogen biotech), 2 mm L‐glutamine, penicillin (100 U mL^−1^), and streptomycin (100 µg mL^−1^). The HeLa S3 *G9a* knockout (*G9a* ^−/−^) cell line was maintained in a medium containing puromycin (0.5 µg mL^−1^). Cells were cultured at 37 °C incubator with 5% CO_2_. Transfections were performed using lipofectamine 2000 according to the manufacturer's instruction. Unless indicated, HEK293T cells were used in most of cell experiments.

### Plasmid Construction

Human G9a (hG9a, Addgene #33025) was subcloned into a pCS2‐3× Flag, pCS2‐3× HA, pCW‐3× Flag, or pGEX‐6P‐1 vector. Human *PPP3CA, PPP2CB, PPP6C, PPP1CC, and PPP2R2A* genes were PCR‐amplified and subcloned into the pCS2‐3× Flag‐N‐ vector. Site‐directed point mutations and Human G9a‐ΔSET were generated using the QuickChange Site‐Directed Mutagenesis protocol (Stratagene), and mutations were confirmed by DNA sequencing. For the luciferase assay, genomic DNA was prepared and the *UHRF1* promoter region (−1921 to +145) was inserted into the 5× *GAL4*‐TATA‐luciferase (Cat. 46756, Addgene), and hG9a and various mutants were subcloned into a pcDNA3‐*GAL4*‐HA‐vector (Cat. 24887, Addgene).^[^ ^55^, ^56^ ^]^

### RNAi Transfection

For knockdown of the indicated genes below, the siRNA sequences are: si*PLK1* 1# 5’‐GGGCGGCTTTGCCAAGTGCTT‐3’, si*PLK1* 2# 5’‐AGATTGTGCCTAAGTCTTT ‐3’, si*PPP3CA*: 5’‐AUAUACGCGUUCUGAAUACTT‐3’,^[^ ^57^ ^]^ si*PPP2CB*: 5’‐GUUCUUCUUGG GAGUAUGUTT‐3’,^[^ ^58^ ^]^ si*PPP6C*: 5’‐UUCGAUCAUGGUCUUCAAATT‐3’,^[^ ^59^ ^]^ si*PPP1CC*: 5’‐CA UCGACAGCAUUAUC CAATT‐3’,^[^ ^60^ ^]^ si*Control*: 5’‐UUCUCCGAACGUGUCACGUTT‐3’. Cells were harvested 48 – 96 h after transfection and analyzed by immunoprecipitation and Western blot.

### Antibodies and Reagents

The following antibodies and reagents were obtained from commercial sources: a‐Flag antibody (F7425‐2MG) were purchased from Sigma‐Aldrich; a‐G9a/EHMT2 (C6H3, #3306S, D5R4R, #68851), a‐Plk1 (4513S) and a‐Cyclin E1(20808) antibodies were purchased from Cell Signaling Technology; a‐cyclin B1 (1495‐1), a‐histone H3 pS10 (1173‐1) were purchased from Epigenomics; a‐pan‐phosoho‐(Ser/Thr) (ab17464), a‐H3K9me1(ab8896), a‐H3K9me2 (ab1220), and a‐H3K9me3 (ab8898) antibodies were purchased from Abcam; a‐GFP (50430‐2‐AP), a‐Myc (60003‐2‐Ig), and a‐Actin (60008‐1‐Ig) antibodies were purchased from Proteintech; a‐PPP2CB (A3122), a‐PPP3CA (A1063), a‐phospho‐PPP2CA/PPP2CB‐Y307 (AP0927), a‐H3K9me2 (A2359) and mouse a‐GAPDH (glyceraldehyde phosphate dehydrogenase) (AC002) antibodies were purchased from ABclonal; a‐H3 (39163) antibody was purchased from Active Motif; a‐PPP6C (HA720056), a‐PPP1CC (500L38), a‐HA (0906‐1) antibodies was purchased from HuaBio; a‐HRP‐Streptavidin antibody (A0303) were purchased from Beyotime; a‐pT1045‐G9a antibody was generated by Proteintech by immunizing rabbits with a pT1045 peptide (RLQLYRpTAKMGW) conjugated with keyhole limpet hemacyanin (KLH); secondary horseradish peroxidase–conjugated mouse or rabbit antibodies were purchased from Jackson ImmunoResearch Laboratories; a‐FLAG M2 affinity gel (A2220), thymidine (T9250), puromycin (P9620) and nocodazole (M1404) were purchased from Sigma‐Aldrich; the hemagglutinin (HA) affinity gel (SA063001) was purchased from Smart‐Lifesciences; BIX‐01294 (S8006) and BI2536 (S1105) was purchased from Selleck; G418 (E856) were purchased from Biosharp.

### Immunoprecipitation and Western Blot

For immunoprecipitation (IP) experiments, cells were collected and lysed in IP buffer [25 mM Tris‐HCl (pH 7.4), 150 mM NaCl, 5% glycerol, 1 mM EDTA, and 1% NP‐40] containing a protease inhibitor cocktail for 30 min on ice. The Flag resin or HA resin was added into the lysates and incubated at 4 °C for 4 h or overnight with rotation. After washed three times with IP buffer, the resins were resuspended into 2× SDS sample buffer. For examine protein levels, cell pellets were added with 2× SDS sample buffer and boiled, and centrifuged. Supernatants were subjected with SDS–polyacrylamide gel electrophoresis (SDS‐PAGE), transferred to nitrocellulose membrane, and immunoblotted with the indicated antibodies.

### Cell Synchronization

HeLa or HeLa S3 cells were synchronized at the G1/S boundary by a double thymidine (2 mm) treatment (18 h of thymidine arrest, 8 h of fresh medium release, followed by an additional 18 h of thymidine arrest) and released. Cells were synchronized at prometaphase by a thymidine‐nocodazole arrest, 18 h of thymidine arrest, and 4 h of fresh medium release, followed by 12 h of nocodazole arrest (100 ng mL^−1^)]. Mitotic cells were then collected by a shake‐off method. HEK293T cells were synchronized at mitotic phase by a nocodazole arrest 18 h.

### TurboID Assay

Flag‐BirA‐hG9a moiety immunoprecipitated from HEK293T cells treatment with or without biotin (100 µm, 16 h), accompanied with 18 h Noc treatment. Cells were collected and lysed in IP buffer [25 mm Tris‐HCl (pH 7.4), 150 mm NaCl, 5% glycerol, 1 mm EDTA, and 1% NP‐40] containing a protease inhibitor cocktail (Cat. B14001, Bimake) for 30 min on ice. The streptavidin resin was added into the lysates and incubated at 4 °C for overnight with rotation. After washed three times with 500 mM NaCl IP buffer, the resins were resuspended into 2× SDS sample buffer, boiled, and centrifuged. Samples were separated by SDS‐PAGE gel, followed by Coomassie blue staining. The whole lane was sent to analysis by MS.

### Mass Spectrometry (MS) Analysis

To identify phosphorylation sites of G9a, GST‐hG9a‐SET (2 µg) was incubated with GST‐N‐Plk1 (0.5 µg) at 30 °C for 1 h in the kinase reaction buffer [25 mm Tris‐HCl (pH 7.5), 0.01% Triton X‐100, 10 mm MgCl_2_, 0.5 mm Na_3_VO_4_, 2.5 mm dithiothreitol (DTT), and 0.5 mm EGTA] in the presence of 1 mm ATP (A600020, Sangon Biotech). Reactions were quenched with 2× SDS sample buffer and run in SDS‐PAGE, followed by Coomassie brilliant blue (CBB) staining. The band corresponding to GST‐hG9a‐SET was subjected to in‐gel trypsin digestion and desalted with C18 tips. The samples were analyzed by liquid chromatography–tandem mass spectrometry (LC‐MS/MS) on a Q Exactive‐HF mass spectrometer (ThermoFisher Scientific). The LC‐MS/MS data were processed using Proteome Discoverer and searched against the Swiss‐Prot *Homo sapiens* protein sequence database.

### Molecular Dynamics (MD) Simulations and Molecular Docking

The crystal structure of G9a‐SET domain in complex with SAM (PDB ID: 3RJW) was obtained from the NCBI database. Structures were analyzed by PyMol software. Different angles of structures containing SAM and local G9a structures were displayed. The residues surrounding SAM were highlighted.

Five models (WT, pT1045, T1045E, T1045A, and T1045S) were constructed for MD simulations. First, these models were processed by adding the missing hydrogen atoms. Then, they were solvated in 80×80×80 Å3 water boxes. Finally, Cl^−^ ion was added to make the systems neutral, and the resulting systems were then energy‐minimized and simulated using the AMBER software package with the AMBER‐ff14SB force field in the periodic boundary conditions. For each system, simulation was performed for ≈100 ns each time. The last 50 ns of each simulation were taken for analysis (the trajectory was sampled every 200 ps). Molecular docking was conducted using Discovery Studio software.

### Protein Expression and Purification

BL21 (DE3) pTf16–competent cells bearing a plasmid encoding GST–hG9a‐SET was induced by 0.1 mm IPTG and L‐arabinose (0.5 mg mL^−1^) at 16 °C for 8 h. Cells were lysed in buffer containing 50 mm Tris‐HCl, 150 mm NaCl, and 0.05% NP‐40, and proteins were purified using a glutathione‐Sepharose resin, followed by elution with glutathione [20 mm in 75 mm Tris‐HCl (pH 8.0)] and dialyzed with PBS buffer.

### Sequential In Vitro Phosphorylation/Methylation Assays

Recombinant GST‐tagged N‐Plk1(0.5 µg) was incubated with GST–hG9a‐SET or hG9a moiety immunoprecipitated from cells in the presence of 1 mm cold ATP and reacted for an additional 1 h. After that, a half volume of reaction mixture was examined by Western blotting to verify the phosphorylation status of G9a. The other half of mixture was added with 1 µg recombinant histone H3 in the presence of 1 mm SAM at 30 °C for 1 h or overnight in the buffer [50 mm Tris‐HCl (pH 8.0), 10% glycerol, 20 mm KCl, 5 mm MgCl_2_, and 1 mm phenylmethyl sulfonyl fluoride]. Reactions were quenched with 5× SDS sample buffer and examined by Western blotting.

### In Vitro Methylation Assay

For protein methylation assays, 1 µg aliquot of bacterially expressed various recombinant GST‐hG9a‐SET proteins were individually incubated with recombinant histone H3 at 30 °C overnight, in the presence of 0.5 µCi of ^3^H‐labeled S‐adenosyl‐methionine or cold SAM. Reactions were spotted onto Whatman P81 filters and washed four times with 50 mm NaHCO_3_ (pH 9.0) before scintillation counting. Alternatively, reactions were quenched by adding an equal volume of 2× SDS sample buffer. Radioactive labeled proteins were detected by CBB staining or Western blotting.

### In Vitro Kinase Assay

Flag‐hG9a moiety immunoprecipitated from HEK293T cells or 1 µg aliquot of bacterially expressed recombinant GST‐ hG9a‐SET was incubated with GST‐N‐Plk1 protein at 30 °C for 1 h in the kinase reaction buffer containing 1 mm cold ATP or 0.5 µCi of γ‐[^32^P] ATP. Reactions were spotted onto a nitrocellulose membrane and immunoblotted with the indicated antibodies. Alternatively, reactions were quenched with 2× SDS sample buffer and run in SDS‐PAGE, followed by CBB and autoradiography.

### Luciferase Reporter Assay

HEK293T cells were co‐transfected with 0.2 µg 5× UAS‐*UHRF1*‐reporter, 1 µg of pcDNA3.1‐based various hG9a plasmids or empty vector, and 0.5 µg Renilla luciferase vector (pRL‐CMV; Promega). After transfection for 48 h, luciferase activities were determined by using the Dual‐luciferase Reporter Assay kit according to the manufacturer's instructions. Renilla luciferase activity was used for normalization of transfection efficiency.^[^ ^23^ ^]^

### RNA‐Sequencing (RNA‐Seq) and Data Analysis

RNA‐seq libraries were prepared using 1 µg of total RNA. Poly‐adenylated RNA isolation, cDNA synthesis, end‐repair, ligation of the Illumina indexed adapters, and libraries amplification were finished by Fast RNA‐seq Lib Prep Kit V2. Libraries were selected for 200 – 300 bp cDNA fragments using VAHTS DNA Clean Beads and were sequenced on the Illumina NovaSeq 6000 v1.5 reagents (pair end). Libraries passing quality control were trimmed by Trim galore (v0.6.7) and mapped to the human genome hg38 using Hisat2 (v7.5.0). The reads counting was used featureCounts (v2.0.1). DESeq2 (v1.34.0) was used to compute differential gene expression using raw read‐counts as input. Heatmaps were generated using the pheatmap package (v1.0.12) in R (4.1.2).

### ChIP‐Sequencing (ChIP‐Seq) and Data Analysis

Cells were cross‐linked for 10 min in 1% formaldehyde and subsequently quenched for 5 min with 0.125 m glycine. Cells were washed two times with cold PBS and lysed in cell lysis buffer [50 mm PIPES (pH 8.0), 85 mm KCl, 0.5% NP‐40] for 20 min on ice, followed by incubation in nuclei lysis buffer [50 mm Tris‐HCl (pH 8.0), 10 mm EDTA, 1% Triton X‐100] at 4 °C for 20 min. Cell sonication was performed using a Bioruptor (Minichiller 300, Diagenode) with 30 cycles (30‐s on, 30‐s off). Chromatin fractions were quantified using the Equalbit dsDNA HS Assay Kit on a Qubit instrument (ThermoFisher Scientific). Approximately 4 – 10% *Drosophila* genomic DNAs were added to samples as a spike‐in control. 31.2 µg of chromatin of each ChIP samples were incubated with 2 µg of H3 or H3K9me2 antibody along with ChIP‐grade protein A/G magnetic beads (26162, Thermo Scientific) at 4 °C overnight, respectively. Beads were washed four times with washing buffer (250 mm LiCl, 10 mm Tris‐HCl, 1 mm EDTA, 1% NP‐40, 1% sodium deoxycholate), and twice with Elution buffer (10 mm NaHCO_3_, 1% SDS) before being de‐crosslinked with proteinase K (1.5 mg mL^−1^) overnight at 65 °C. DNA was extracted using the Universal DNA Purification Kit. ChIP‐seq libraries were generated using the VAHTSTM Universal DNA Library Prep Kit for Illumina® V3 and sequenced according to the NovaSeq 6000 protocol.

Sequencing adapters were trimmed by Trim galore (v0.6.7). *Drosophila melanogaster* genome was used as a spike‐in control for ChIP‐seq. The chimeric *H. sapiens‐Drosophila* genome (GRCh38‐ GCF_000001215.4) was generated by STAR (v2.7.10a) with the genomeGenerate tool.^[^ ^61^ ^]^ The chimeric genome contains sequence data of these two species. The clean reads were mapped to chimeric genome using STAR with the parameter –runThreadN 16 –runMode alignReads– readFilesCommand zcat –quantMode TranscriptomeSAM GeneCounts –twopassMode Basic –outSAMtype BAM Unsorted –outSAMunmapped None. The data splitting and sorting were finished by SAMtools (v1.3.1). The BEDtools (v2.30.0) were used for the generation of BigWig files and the normalizations of those spike‐in signals.^[^ ^62^ ^]^ Deeptools (v3.5.1) was used to visualize the H3K9me2 signals after normalization. Peaks were called by Macs2 (v2.1.1). Deeptools (v3.5.1) was used to plot the signal of these BigWig files. The intergenic region location was obtained by BEDtools intersect (v2.30.0). Different peaks were defined by DiffBind (v3.4.11) (FDR (false discovery rate) ≤ 0.05).

### ATAC‐Sequencing (ATAC‐Seq) and Data Analysis

ATAC‐seq analysis was performed as described previously with some modifications.^[^ ^63^ ^]^ In brief, 50 000 HeLa S3 cells were lysed with buffer [10 mm Tris‐HCl (pH 7.4), 10 mm NaCl, 3 mm MgCl_2_, 0.1% (v/v) NP‐40]. The lysate was centrifuged at 500 g for 5 min at 4 °C. Nuclei was treated with Hieff NGS Fast Tagment DNA Library Prep Kit for Illumina (Cat. 12207, Yeasen) for 30 min at 37 °C. DNA fragments were purified by VAHTS DNA Clean Beads and amplified by PCR following manufacturer manuals. The amplified library was further purified by DNA Clean Beads.

ATAC‐seq libraries were sequenced on the Illumina NovaSeq 6000 v1.5 reagents (pair end). The raw reads were removed adapters by Trim galore (v0.6.7) (‐q 20 –phred33 –length 30 ‐e 0.1 –stringency 4). The clean reads were mapped to the hg38 using Bowtie2 (v2.3.5.1). The bam files should be sorted by SAMtools (v1.3.1), and PCR replication removed by Sambamba (v0.8.1). Reads mapped to the chromosome M and low quality of reads (samtools view ‐q 30) were discarded. Narrow peaks were called by Macs2 (v2.1.1) using the parameter (–nomodel –shift ‐100 –extsize 200). Deeptools (v3.5.1) was used to plot heatmaps and profiles. Browser tracks were visualized by IGV Browser (v2.11.4) after normalizing the reads with RPKM (Reads Per Kilobase per Million mapped reads). DiffBind (v3.4.11) was used to analyze the different peaks in different samples (FDR (false discovery rate) ≤ 0.05). The different peaks were annotated to the gene location.

### Dot Blot Assay

Aliquots(1.5 µL) of samples were spotted on nitrocellulose membrane. Air dried membrane was stained by ponceau S. Membranes were soaked in 5% skimmed milk in 1× TBS‐T (10 mm Tris, 150 mm NaCl, pH 8.0, Tween‐20 0.1%) buffer at room temperature for 40 min to block non‐specific binding. The membrane was then incubated with the primary antibody at 4 °C for 1.5 h or overnight. The membrane was washed with 1 × TBS‐T and then incubated with the secondary antibody at room temperature for 1 h and washed with 1 × TBS‐T. The chemiluminescent signals were detected using super enhanced chemiluminescence detection reagent and quantified by ChemiScope 3300 Mini (CLiNX).

### In Vitro Phosphatase Assay

Aliquot(10 µg) of pT1045 peptide were incubated with the indicated phosphatase proteins immunoprecipitated from HEK293T cells at 30 °C for 15 min in the buffer [20 mm Tris‐HCl, pH 7.4, 1% Triton X‐100, 250 mm sucrose, 1 mm MnCl2, and 0.1% β‐mercaptoethanol].^[^ ^64^ ^]^ Samples were spotted onto nitrocellulose membranes and immunoblotted with an a‐pT1045‐G9a antibody.

### Flow Cytometry

Cells were harvested at different times after double‐thymidine release, washed twice with PBS, fixed using 70% ethanol overnight at −20 °C, stained with propidium (20 µg mL^−1^) in the presence of RNase A (200 µg mL^−1^) and 0.1% Triton X‐100 for 30 min, and then 20 000 single cells from each sample were counted and analyzed by the BD FACSAria system (BD Biosciences). Data were analyzed by FlowJo data analysis software.

### Immunofluorescence Microscopy

HeLa S3 cells were cultured on glass coverslips, washed with PBS two times, fixed with 4% paraformaldehyde for 10 min at room temperature, and permeabilized with 0.3% Triton X‐100 for 30 min at 37 °C. Then, cells were blocked with 3% BSA in PBS and incubated for 30 min at room temperature or overnight at 4 °C. Cells were then incubated with the indicated primary antibody at room temperature for 2 h or at 4 °C overnight. After washed with PBS three times, cells were incubated with the appropriate secondary antibody at room temperature for 1 h. The coverslips were stained with 4’,6‐diamidino‐2‐phenylindole (DAPI) and mounted. Immunofluorescence images were captured under a confocal laser scanning microscope (Leica SP8).

### Quantification and Statistical Analysis

For quantification of Western blot, ImageJ software was used to measure the relative intensity of each band, and the relative protein or modification levels were normalized to levels of loading controls. Unless otherwise indicated, data are presented as the means ± SD from at least three independent experiments, and the differences between any two groups were compared by unpaired *t*‐test. The data were scatter‐plotted using the Prism 7 software. *P < 0.05, **P < 0.01, ***P < 0.001, and ns indicates ‘not significant’.

## Conflict of Interest

The authors declare no conflict of interest.

## Author Contributions

Q.G., Y.K., W.L., and J.Z. contributed equally to this work. Q.G., Y.K., and H.‐N.D. conceived the project, Q.G., Y.K., and W.L. conducted experiments, Y.K. performed genomic analysis, J.Z., Y.Z., L.‐T.D., and H.‐N.D. designed and conducted molecular docking, Q.G., Y.K., W.L., J.Z., H.M., Y.Z., and H.‐N.D. acquired and analyzed data. H.‐N.D. wrote the manuscript with inputs from other authors. All authors read and approved the final manuscript.

## Supporting information

Supporting Information 1

Supporting Information 2

## Data Availability

The data that support the findings of this study are available in the supplementary material of this article.
