# Supplementary material for: Dynamic Phosphorylation of G9a Regulates its Repressive Activity on Chromatin Accessibility and Mitotic Progression
Source: Adv Sci (Weinh). 2023 Sep 3;10(30):2303224. doi: 10.1002/advs.202303224 (PMC10602519; doi:10.1002/advs.202303224)
Supplement: Supplementary file 1 — Supporting Information 1 [file ADVS-10-2303224-s002.pdf]

ADVANCED SCIENCE
Open Access

Supporting Information

for *Adv. Sci.*, DOI 10.1002/advs.202303224

Dynamic Phosphorylation of G9a Regulates its Repressive Activity on Chromatin Accessibility and Mitotic Progression

Qizhi Geng, Yue-Yu Kong, Weizhe Li, Jianhao Zhang, Haoli Ma, Yuhang Zhang, Lin-Tai Da, Yan Zhao and Hai-Ning Du**

Supplementary Information

Dynamic phosphorylation of G9a modulates its repressive activity on chromatin accessibility and mitotic progression

Qizhi Geng,[1,†] Yue-Yu Kong,[1,†], Weizhe Li,[1,†], Jianhao Zhang,[2,†], Haoli Ma,[3] Yuhang Zhang,[2] Lin-Tai Da[4], Yan Zhao,[3,*] Hai-Ning Du[1,*]

[1] Hubei Key Laboratory of Cell Homeostasis, College of Life Sciences, Hubei Clinical Research Center of Emergency and Resuscitation, Emergency Center of Zhongnan Hospital, Frontier Science Center for Immunology and Metabolism, RNA Institute, Wuhan University, Wuhan 430072, China

[2] School of Life Sciences and Biotechnology, Shanghai JiaoTong University, Shanghai 200240, China

[3] Hubei Clinical Research Center of Emergency and Resuscitation, Emergency Center of Zhongnan Hospital of Wuhan University, Wuhan University, Wuhan 430071, China

[4] Shanghai Center for Systems Biomedicine, Shanghai JiaoTong University, Shanghai 200240, China

[†] Q. G., Y. K., W.L., and J.Z. contributed equally to this work.

*To whom correspondence should be: Email: hainingdu@whu.edu.cn; or doctoryanzhao@whu.edu.cn

Supplementary Figure S1-10

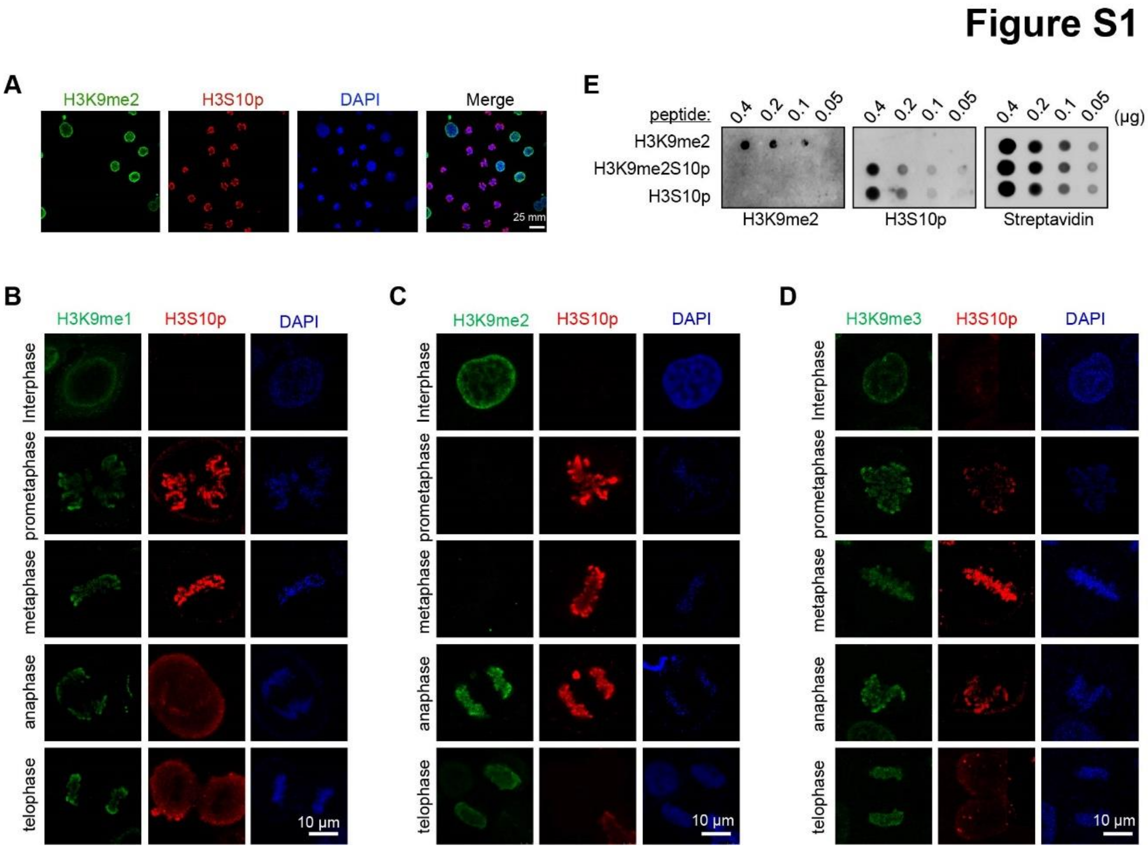

Figure S1. H3K9me2 levels are dynamically changed during cell cycle progression. A) Representative immunostaining images showed that H3K9me2 (green) and phosphorylated H3Ser10 (H3pS10, red) marks are mutually exclusive in individual cells. DNA was stained with DAPI (blue). *Scale bar:* 25 μm. B-D) Representative immunostaining images showed that HeLa cells stained with the indicated H3K9 methyl antibodies (green) and pH3S10 antibody (red) at different mitotic stages from prometaphase to telophase. DNA was stained with DAPI (blue). *Scale bar:* 10 μm. E) Dot blots to examine the specificity of anti-H3K9me2 or anti-H3S10p antibodies using the indicated biotin-tagged peptides. Anti-Streptavidin antibody to examine the loading levels of the peptides.

Figure S2

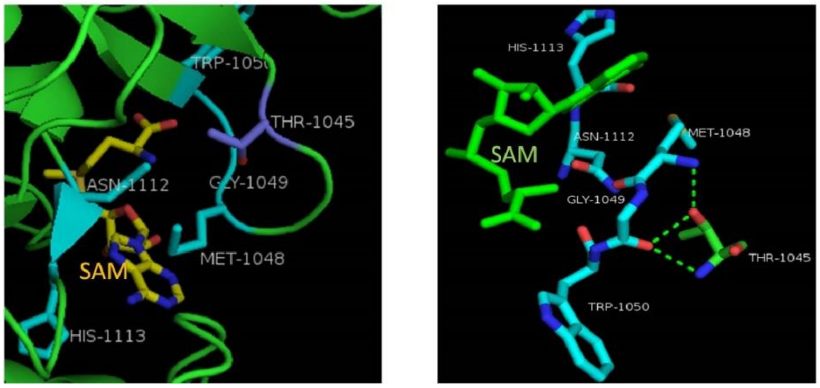

Figure S2. X-ray structures showed local conformation of G9a with SAM. Structures are generated by Pymol based on crystal structure (PDB: 3RJW). Local structure of G9a (green backbone with purple side chain) with SAM (yellow) were presented (left). Hydrogen bonds between T1045 with M1048 and G1049 were present with dot line (right). The amino acids that involve in the interaction with SAM were labelled.

Figure S3

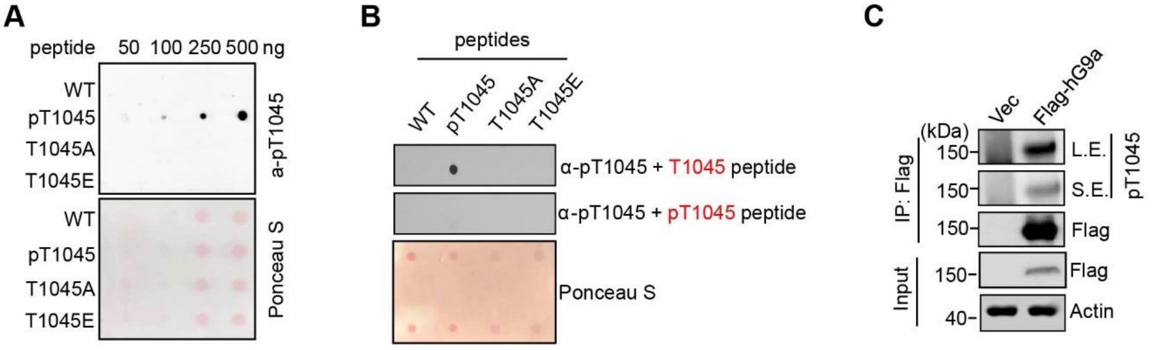

Figure S3. Validation of the specificity of anti-pT1045 antibody. A) Dot blots showed that the anti-pT1045 antibody specifically recognized pT1045 peptide (top panel). The equal amounts of various peptides were examined by Ponceau S staining (bottom panel). B) Peptide blocking experiments to show the anti-pT1045 antibody was specifically recognized by the phosphorylated T1045 peptide but not the non-phosphorylated T1045 peptide or other T1045 mutant peptides. C) Western blot showed that the anti-pT1045 antibody only recognizes Flag-hG9a immunoprecipitated from HEK293T cells, but not empty vector (Vec).

Figure S4

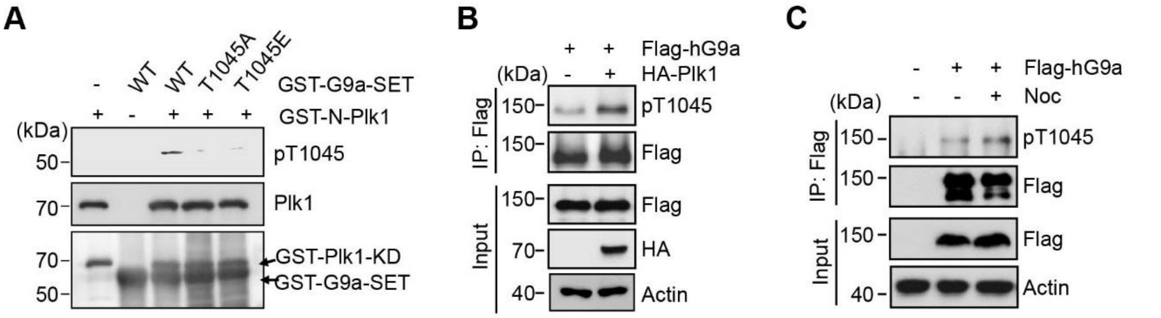

Figure S4. G9a was phosphorylated at T1045 by Plk1 mainly at mitosis. **A)** *In vitro* kinase assay showed that recombinant N-Plk1 protein directly phosphorylates recombinant WT G9a-SET fragment, but not T1045 mutated fragments. **B)** Western blot showed that overexpression of Plk1 in HEK293T cells increased phosphorylation level of T1045. **C)** Western blot showed that nocodazole-treated HeLa cells stably expressing Flag-hG9a exhibited elevated pT1045 level.

Figure S5

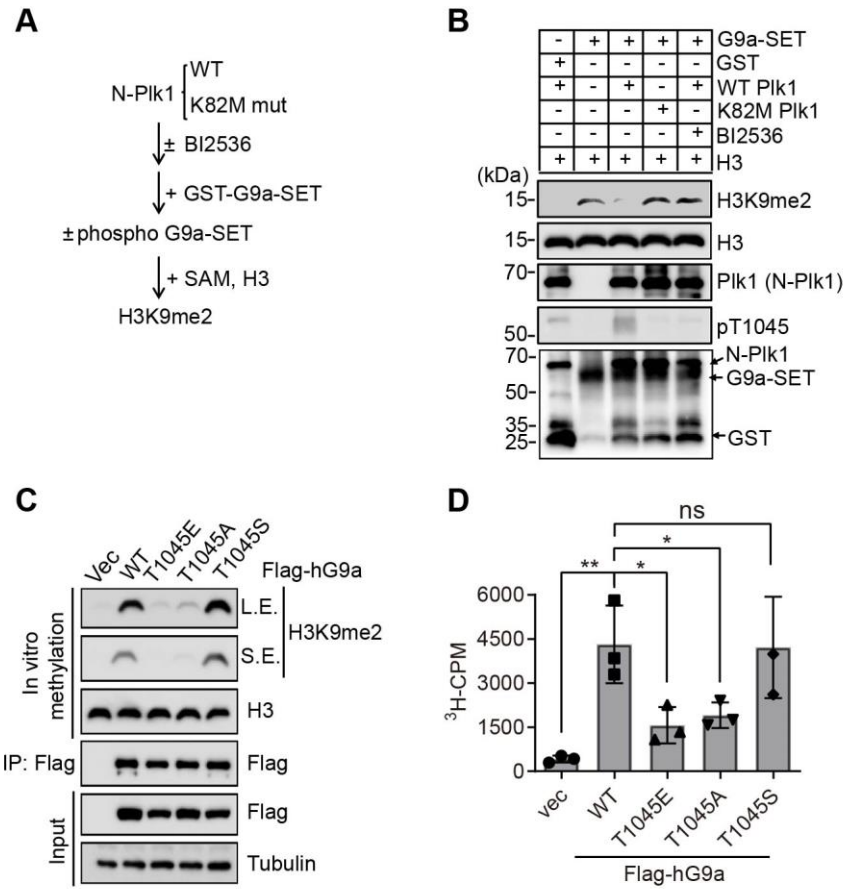

Figure S5. *In vitro* assays showed that Plk1-mediated pT1045 of G9a attenuates its catalytic activity of H3K9me2. A, B) Schematic diagram of sequential *in vitro* phosphorylation-methylation assays (A) were performed in (B) using recombinant WT or K82M mutant N-Plk1 and G9a-SET proteins. The Plk1 inhibitor of BI2536 was used. C, D) *in vitro* methylation assays showed that WT or T1045S mutant, but not T1045A or T1045E mutant of G9a, exhibited catalytic activities towards H3K9me2 in HEK293T cells examined by Western blots (C) and liquid scintillation counting (D). Error bars denote the mean ± SD from three independent experiments. Unpaired *t*-test, * $P < 0.05$, ** $P < 0.01$, ns, not significant.

Figure S6

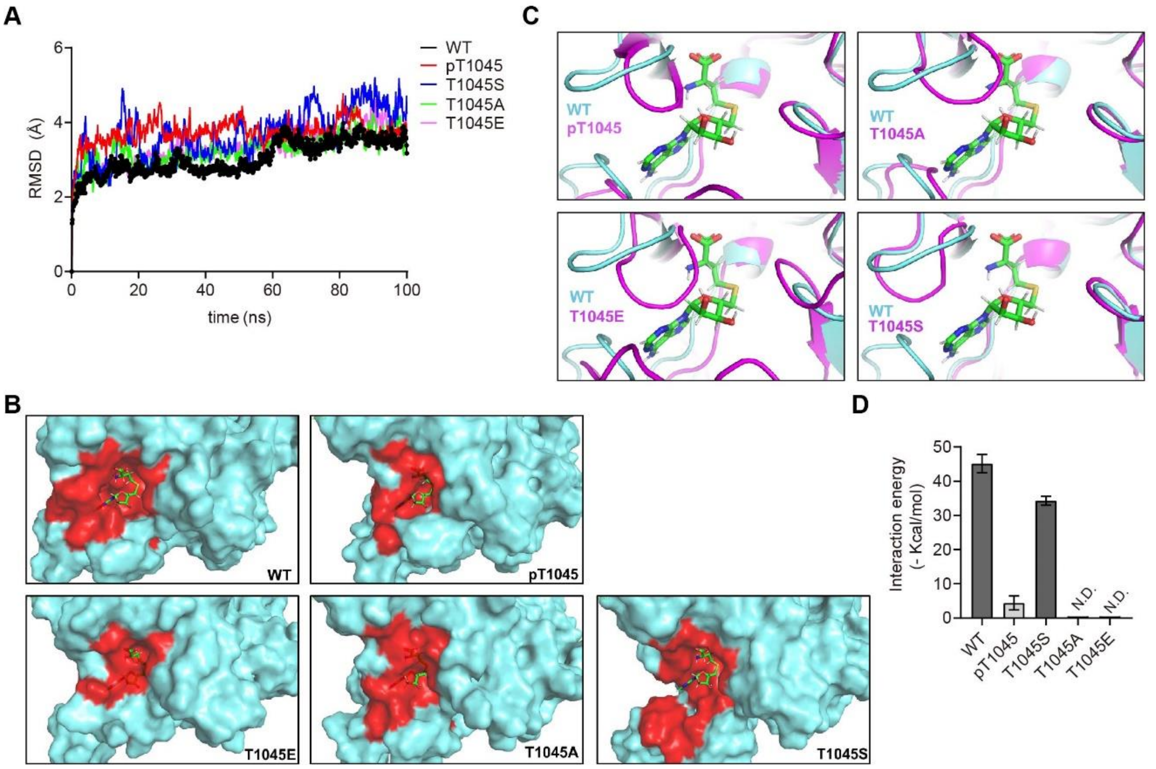

Figure S6. Molecular docking shows that pT1045 and T1045 mutants have weaker binding capacities with SAM. A) The root-mean-square deviation (RMSD) was used to measure structural deformation by T1045 mutants and pT1045 of full-length G9a protein in 100-ns of molecular dynamics (MD) simulations. B) Spacing-filling structural representations of SAM binding pockets (red) of G9a (cyan) generated by MD simulations were docked with a SAM donor (green). Only the binding pockets of WT and T1045S mutant can fit with an integral SAM molecule. C) Superimposed structural representations show the conformational changes introduced by T1045 phosphorylation or various mutants (magenta) compared with the WT G9a (cyan). The conformation of WT G9a (WT) was obtained from the crystal structure (PDB ID 3RJW). Conformation of pT1045 and T1045 mutants are representative snapshots from the MD simulations. D) Molecular docking showed that the average interaction energy of G9a and SAM from the predicted top 10 stabilized conformations. Error bars indicate mean ± SD. N.D. represents ‘not determined’.

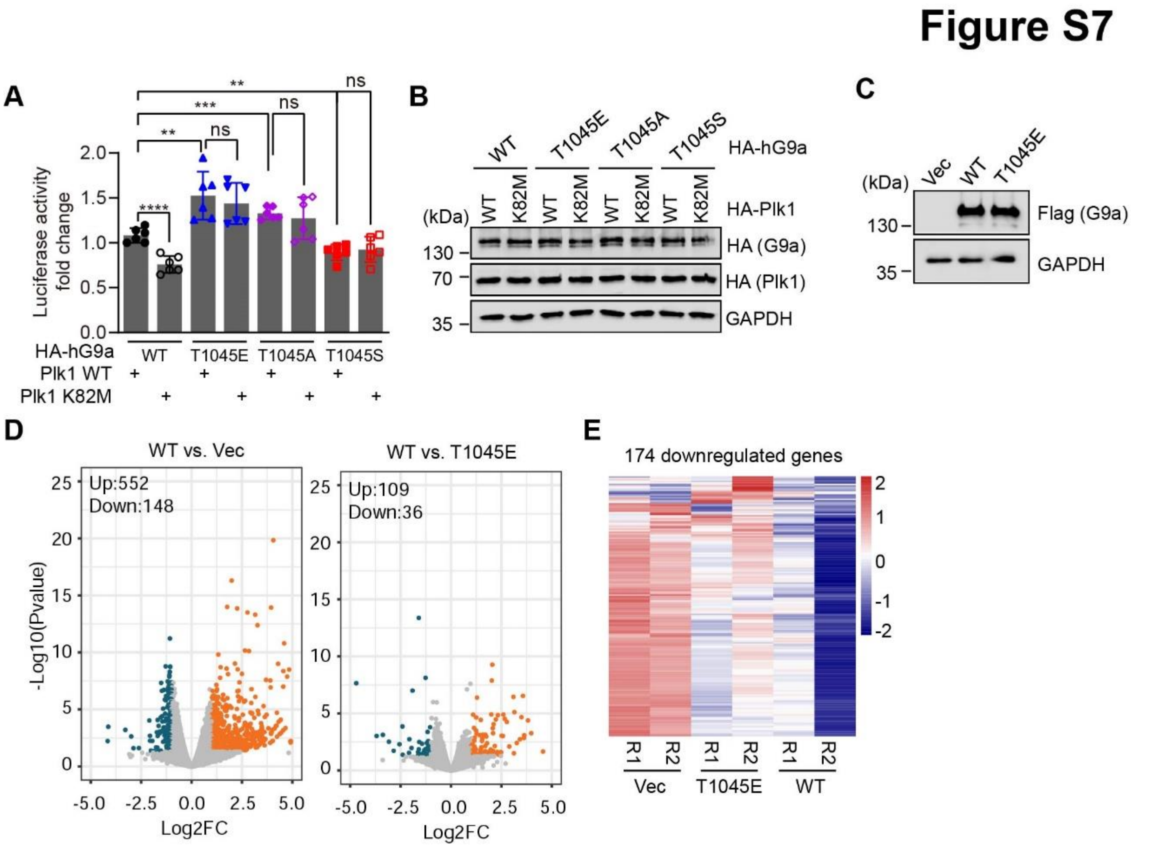

Figure S7. T1045 phosphorylation of G9a attenuated its repressive activity of gene expression. A, B) Luciferase assays showed the effects of WT or K82M mutant of Plk1 on the repressive activity of various G9a constructs. Relative luciferase activity from multiple independent experiments was plotted (A), and protein levels from the indicated samples were examined by Western blots (B). C) Western blots showed protein levels of the indicated G9a constructs used in RNA-seq. D) Volcano plots showed differential gene expression patterns in WT cells relative to cells bearing empty vector (Vec, left panel) or to cells bearing T1045E mutant (right panel). Downregulated genes showed with navy blue, upregulated genes showed with orange. Significance based on DESeq2 analysis with multiple-comparison correction (|Log2FC| >1, P < 0.05). E) Heatmaps depicted expression patterns of downregulated genes in WT cells relative to cells expressing Vec or T1045E (n = 174). Two replicates for each were presented. Error bars denote the mean ± SD from three independent experiments. Unpaired t-test, ** P < 0.01, *** P < 0.001, **** P < 0.0001, ns, not significant.

Figure S8

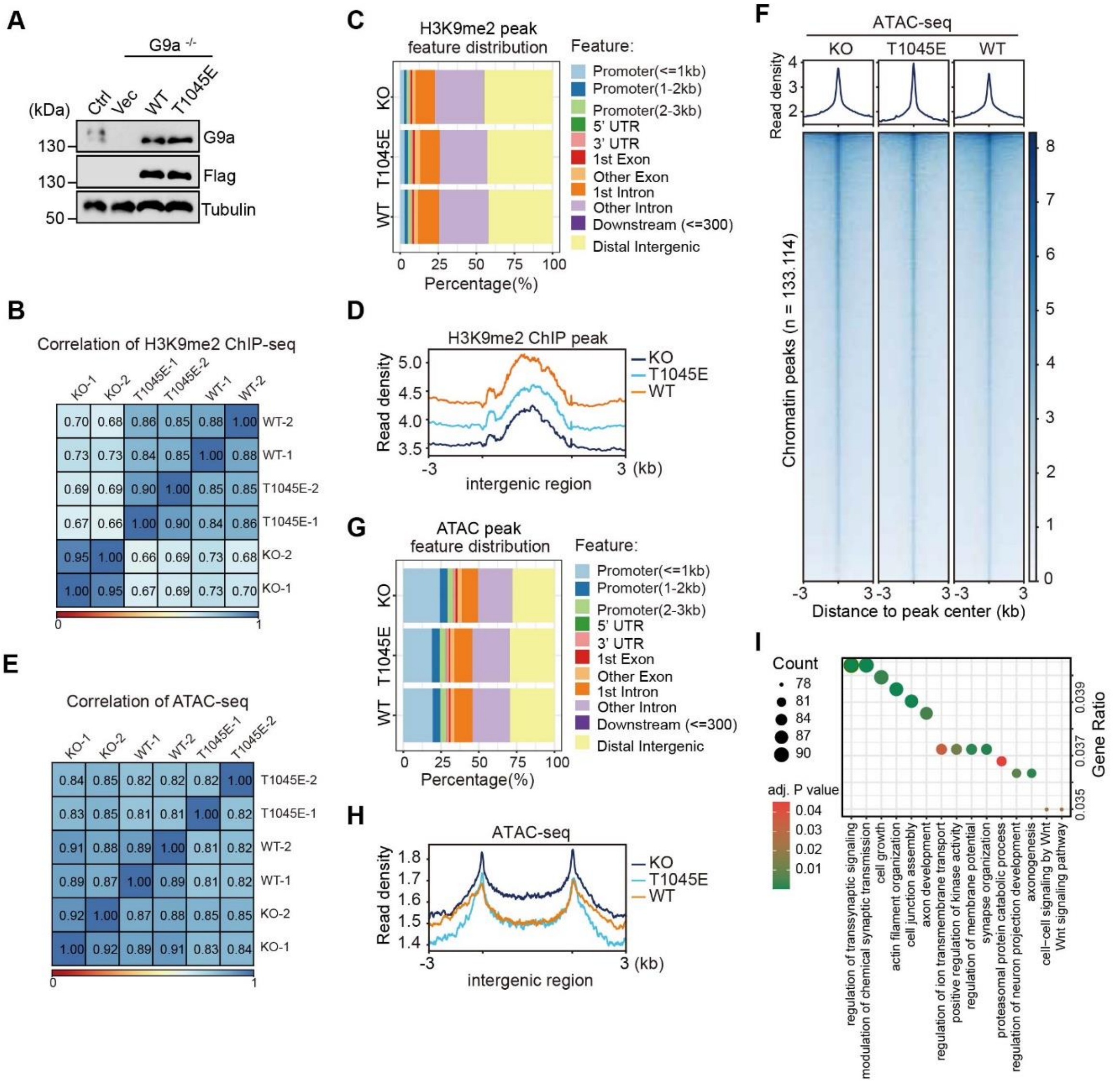

Figure S8. T1045 phosphorylation of G9a reduced H3K9me2 occupancy and increased chromatin accessibility at promoter regions. A) Western blots showed protein levels of WT or T1045E mutant expressed in G9a$^{-/-}$ cells, which were used in ChIP-seq and ATAC-seq. B) Sample distance heatmaps showed the correlation of ChIP-seq samples. The numbers show the Pearson correlation coefficient between each pair of samples with two independent replicates. C) The genomic annotation of H3K9me2 ChIP-seq peaks in the indicated cells. D) Profiles showed that averaged intensities of H3K9me2 ChIP-seq in the intergenic regions of the indicated cells. E) Sample distance heatmaps showed the correlation of ATAC-seq samples. The numbers show the Pearson correlation coefficient between each pair of samples with two independent replicates. F) Averaged signal intensities (top) and heatmaps (bottom) showed the signals of ATAC-seq ± 3 kb from the center of

peaks (*n* = 133,114) in the indicated cells. G) The genomic annotation of ATAC-seq peaks in the indicated cells. H) Profiles showed that averaged intensities of ATAC-seq in the intergenic regions of the indicated cells. I) Gene Oncology analysis of biological processes showed the significant downregulated genes described in Fig. 5F.

Figure S9

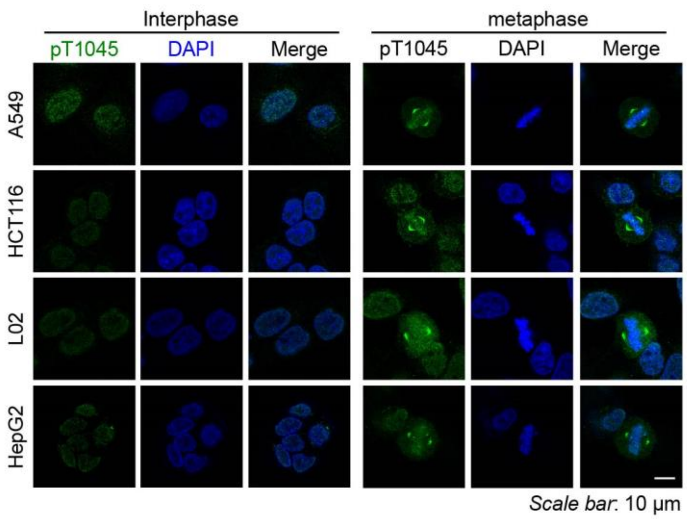

Figure S9. Representative immunostaining images show the signals of pT1045 (green) at interphase (left panel) or metaphase (right panel) from four indicated cell lines. DNA was stained with DAPI (blue).

Figure S10

A

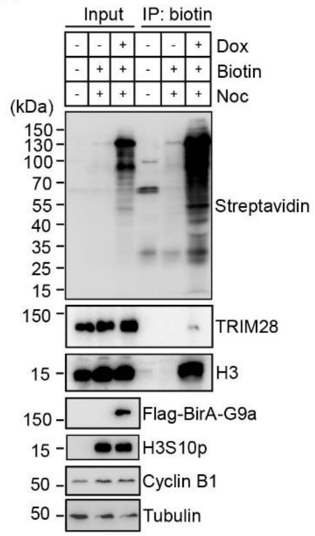

B

Protein name	Peptide No.	Unique peptide No.	Peptide sequences
PPP1CC	19	19	IFCCHGGLSPDLQSMEQIR<br>AHQVVEDGYEFFAKR<br>ICGDIHGQYYDLLR<br>AHQVVEDGYEFFAK<br>IFCCHGGLSPDLQSMEQIRR<br>QSLETICLLLAYK<br>IMRPTDVPDQGLLCDLLWSDPDKDVLGWGENDR<br>EIFLSQPILLELEAPLK<br>TFTDCFNCLPIAAIVDEK<br>SREIFLSQPILLELEAPLK<br>IKYPENFFLLR<br>GKQSLETICLLLAYK<br>YPENFFLLR<br>FLHKHDLDLICR<br>IYGFYDECKR<br>NVQLQENEIR<br>HDLDLICR<br>KKPNATRPVTPPR<br>IYGFYDECK
PPP2R2A	1	1	VPVFRPMDLMVEASPR
PPP2CB	1	1	YSFLQFDPAPR
PPP3CA	2	2	YLFLGDYVDR<br>TLFLLR
PPP6C	1	1	GAGWLFGAK

C

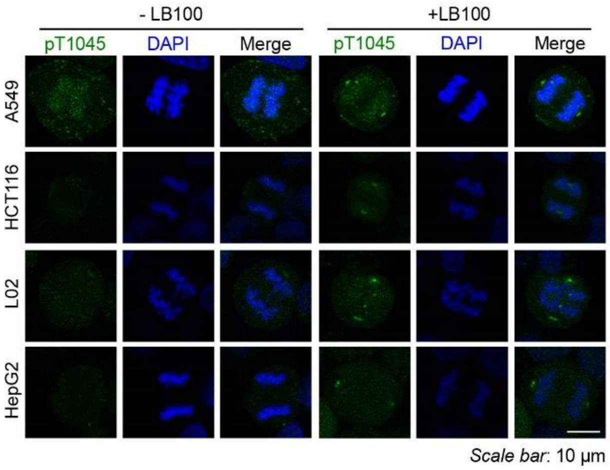

Figure S10. TurboID identified several phosphatases interacting with G9a. A) Biotin-tagged G9a in HEK293T cells was enriched by streptavidin beads, and co-immunoprecipitated proteins were examined by Western blots. Interacting proteins of G9a were identified by mass spectrometry. B) A list of identified phosphatases interacting with G9a. The unique peptides were counted and summarized. C) Representative immunostaining images show the intensity differences of pT1045 signals (green) at late mitosis in the absence or presence of LB100 from four indicated cell lines. DNA was stained with DAPI (blue).
