# Supplementary material for: Dynamic Phosphorylation of G9a Regulates its Repressive Activity on Chromatin Accessibility and Mitotic Progression
Source: Adv Sci (Weinh). 2023 Sep 3;10(30):2303224. doi: 10.1002/advs.202303224 (PMC10602519; doi:10.1002/advs.202303224)

ADVANCED SCIENCE
Open Access

Supporting Information

for *Adv. Sci.*, DOI 10.1002/advs.202303224

Dynamic Phosphorylation of G9a Regulates its Repressive Activity on Chromatin Accessibility and Mitotic Progression

Qizhi Geng, Yue-Yu Kong, Weizhe Li, Jianhao Zhang, Haoli Ma, Yuhang Zhang, Lin-Tai Da, Yan Zhao and Hai-Ning Du**

Figure 1

Fig. 1A

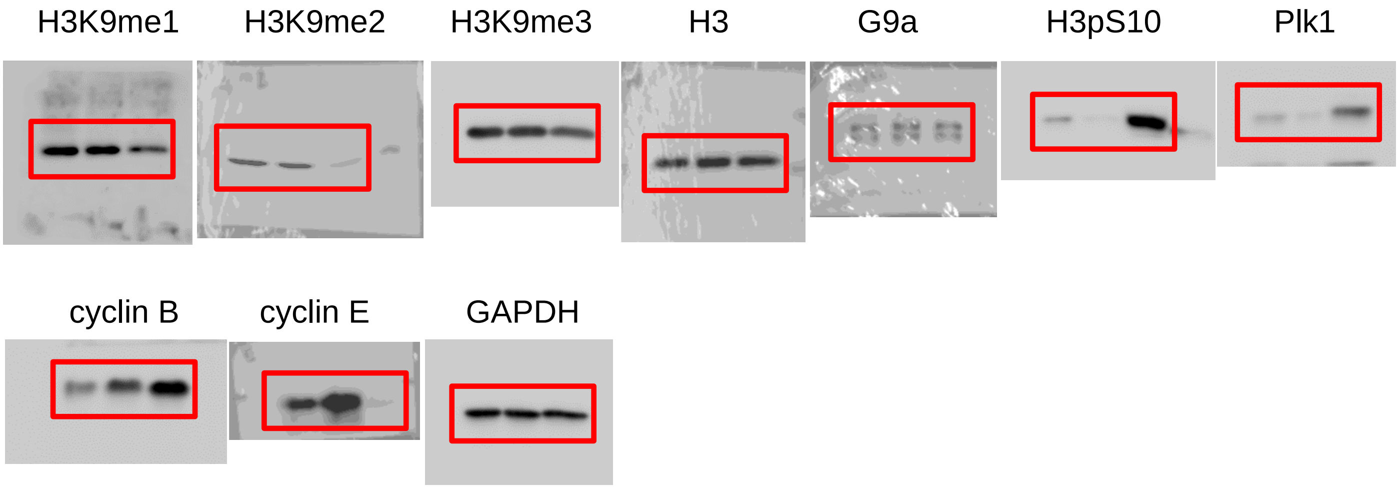

Fig. 1B

	Asyn			G1/S			M		
	Exp 1	Exp 2	Exp 3	Exp 1	Exp 2	Exp 3	Exp 1	Exp 2	Exp 3
H3K9me1/H3	1	1	1	0.833	1.02	0.778	0.496	0.616	0.770
H3K9me2/H3	1	1	1	0.825	0.967	0.747	0.396	0.467	0.884
H3K9me3/H3	1	1	1	1.21	1.28	1.01	0.846	1.086	1.043

Fig.1C

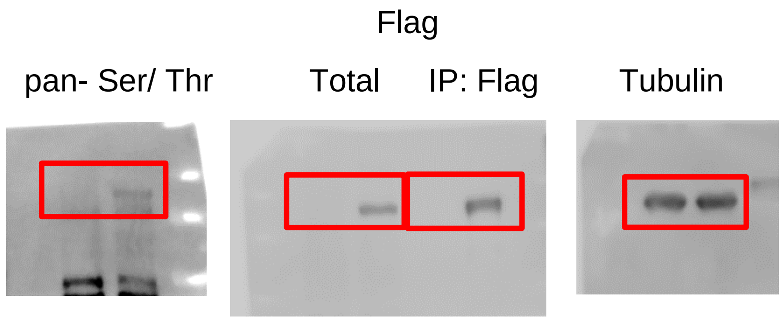

Fig. 1D

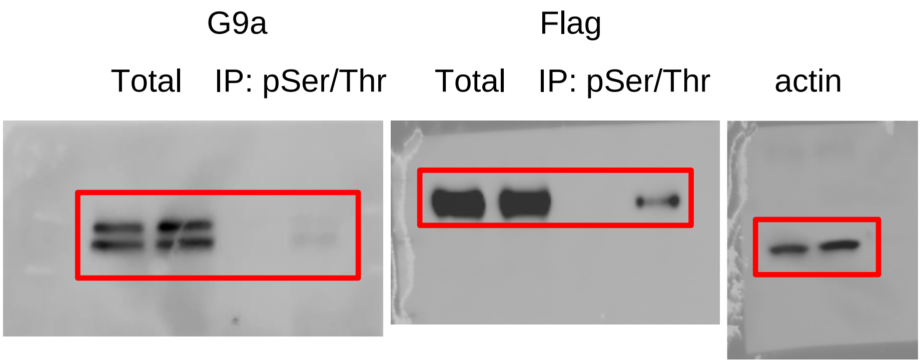

(Figure 1 continued)

Fig. 1E

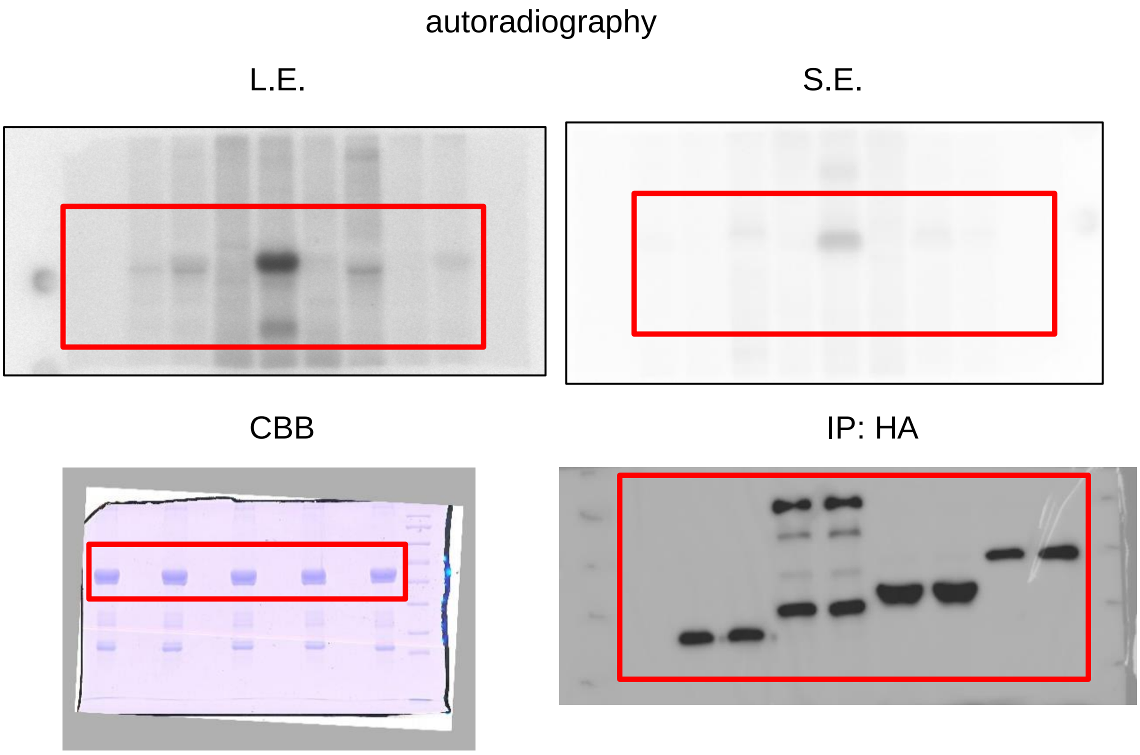

Fig. 1F

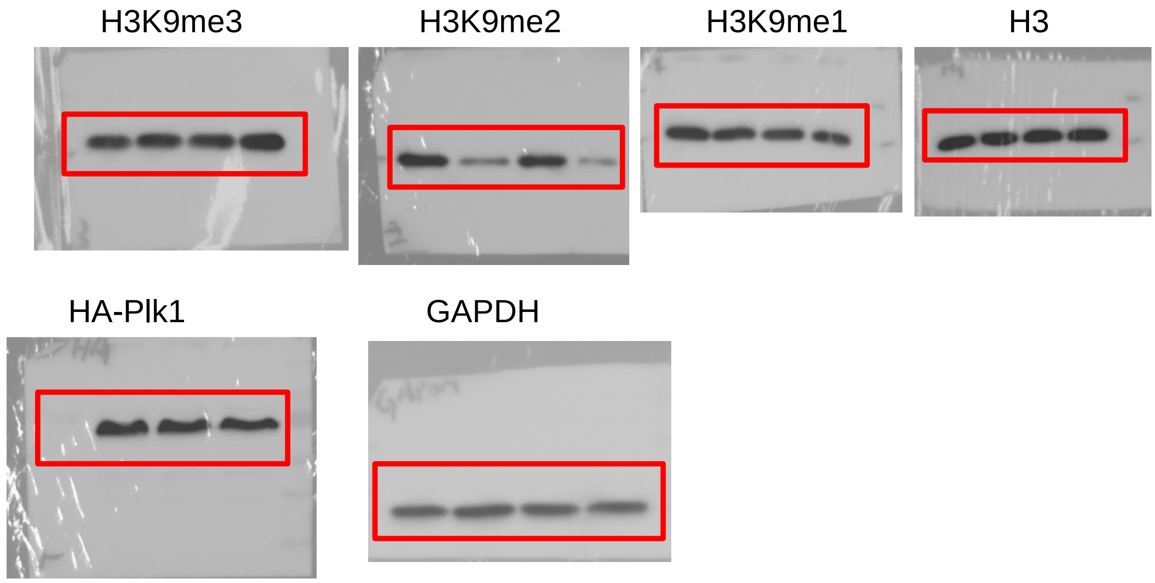

Fig. 1G

HA-Plk1		Vec	WT	K82M	T210D
Exp 1	H3K9me2/H3	1	0.6252423	1.240001	0.6318552
Exp 2	H3K9me2/H3	1	0.3611654	0.7460449	0.3446819
Exp 3	H3K9me2/H3	1	0.6495186	1.019644	0.6572228

(Figure 1 continued)

Fig. 1H

H3K9me3 H3K9me2 H3K9me1 H3 Plk1 GAPDH

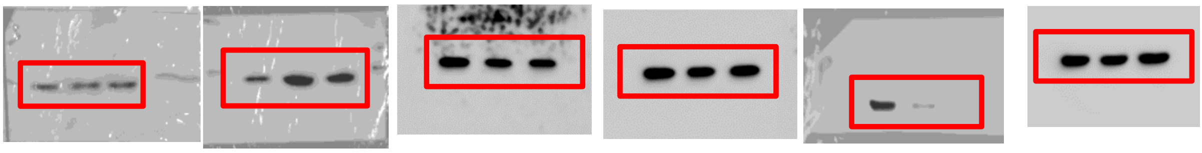

Fig. 1I

		si*NC*	si*PLK1*-1	si*PLK1*-2
Exp 1	H3K9me2/H3	1.000	1.532	1.643
Exp 2	H3K9me2/H3	1.000	1.971	2.139
Exp 3	H3K9me2/H3	1.000	1.765	1.665

Fig. 1J

Autoradiography CBB

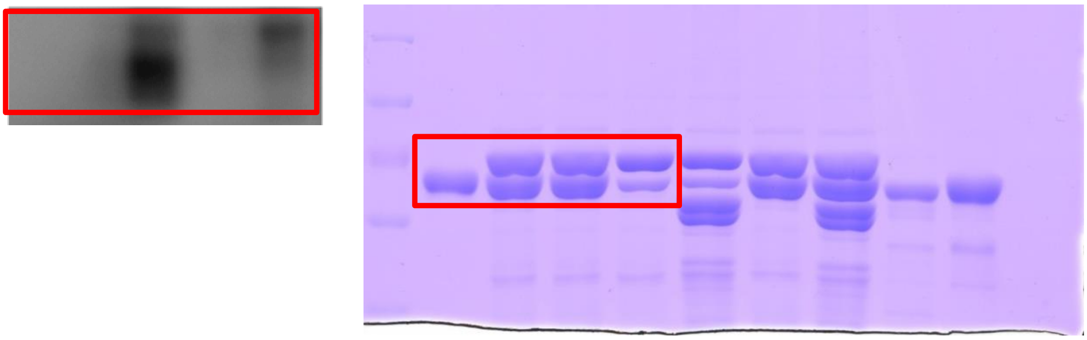

Figure 2

Fig. 2C

Autoradiography CBB

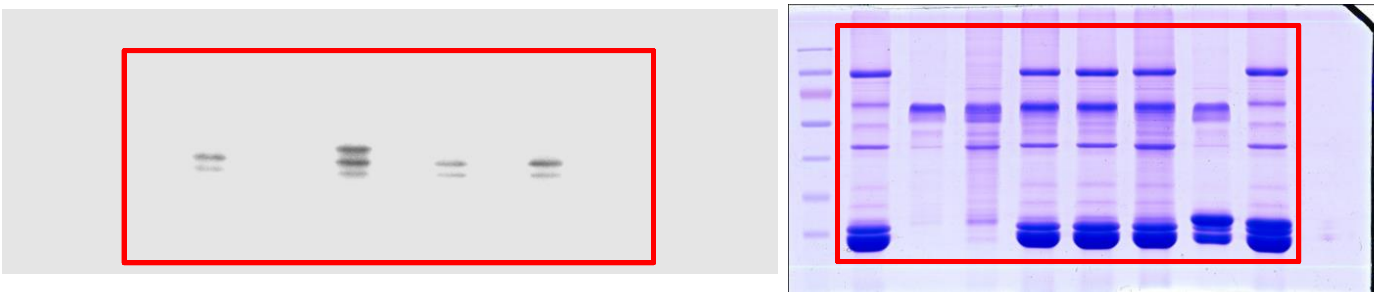

Fig. 2D

G9a

pT1045 Total IP: G9a

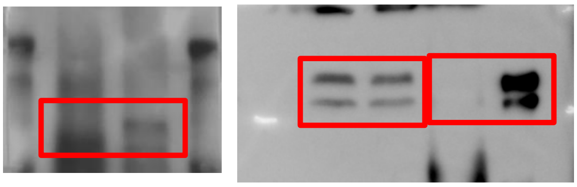

Fig. 2E

pT1045 IP: Flag Total Actin

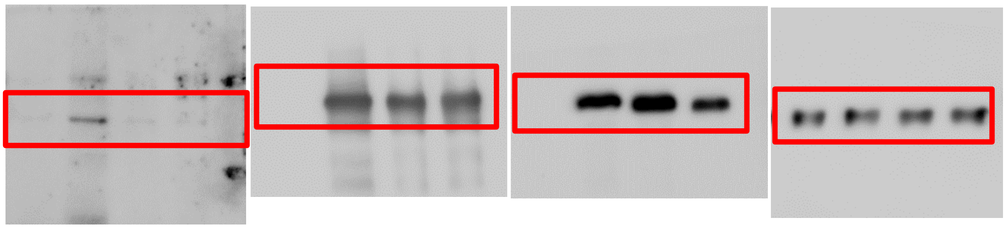

Fig. 2F

pT1045 Plk1 Flag IP: Flag

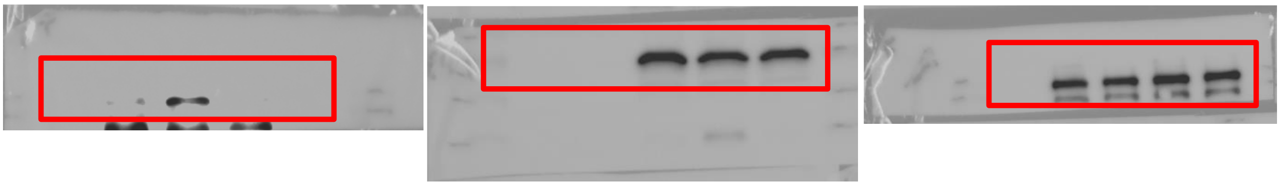

Flag Total Tubulin

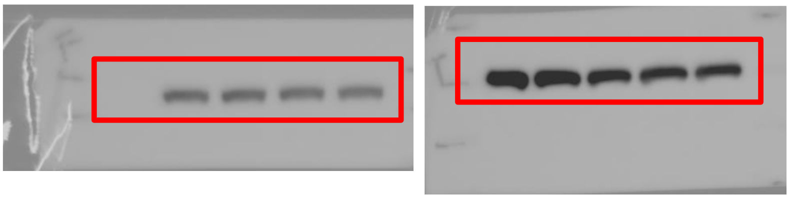

(Figure 2 continued)

Fig. 2G

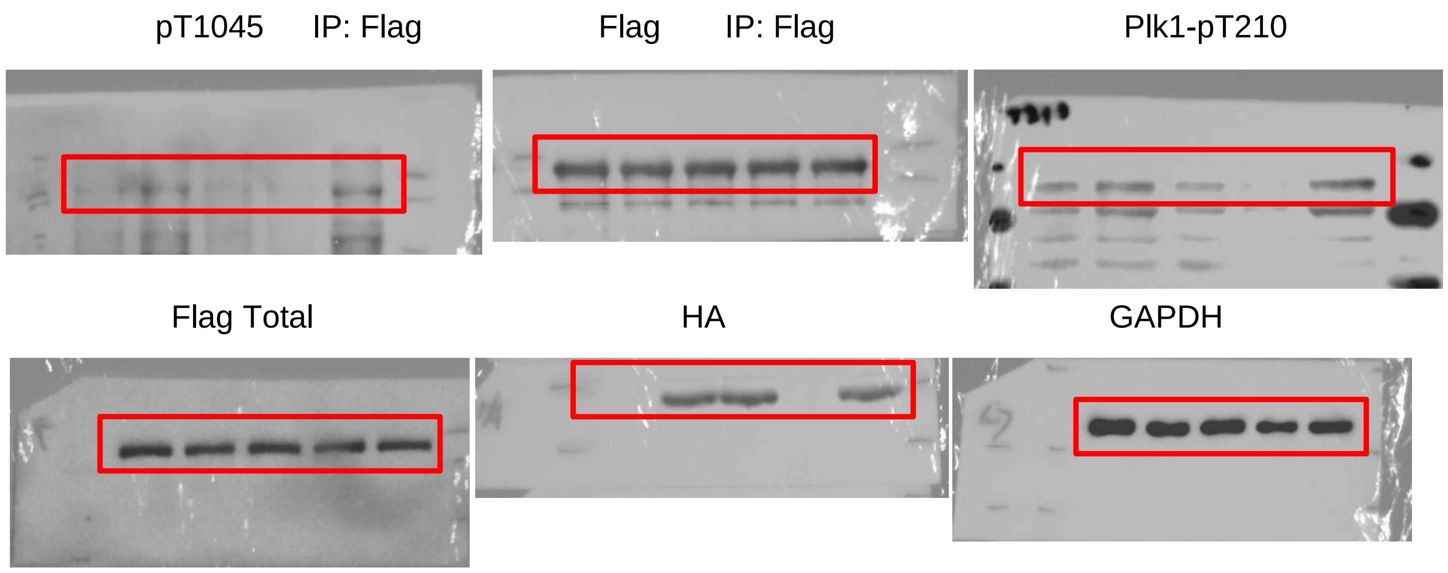

Fig. 2H

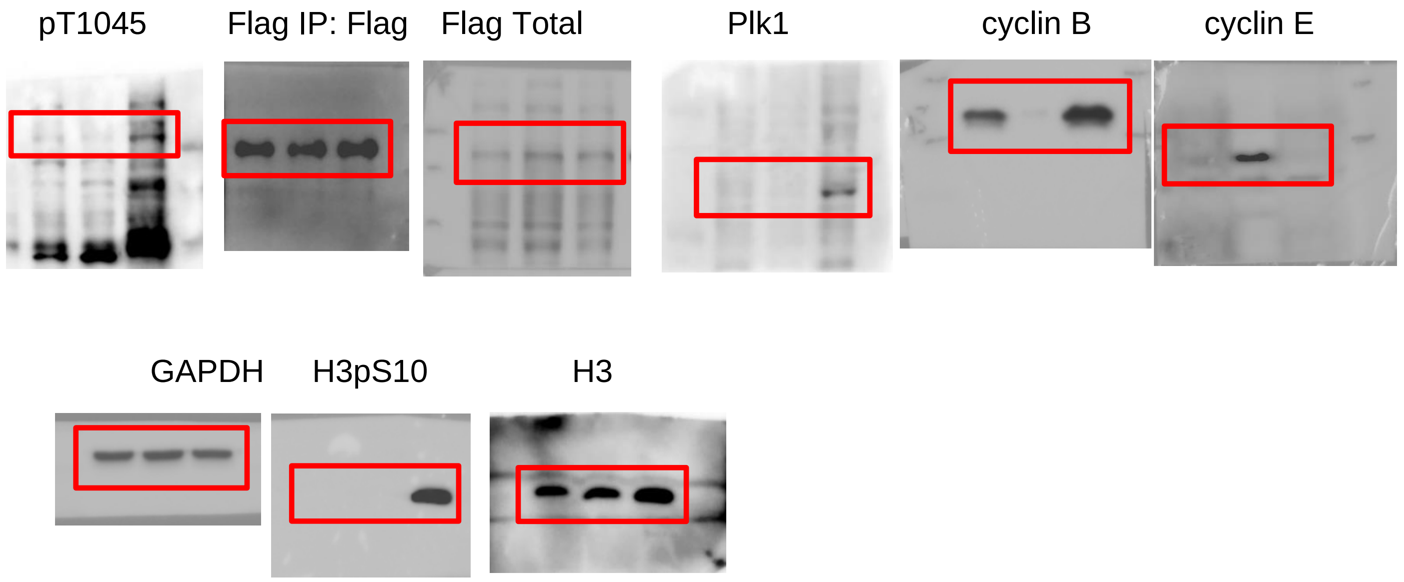

Fig. 2I

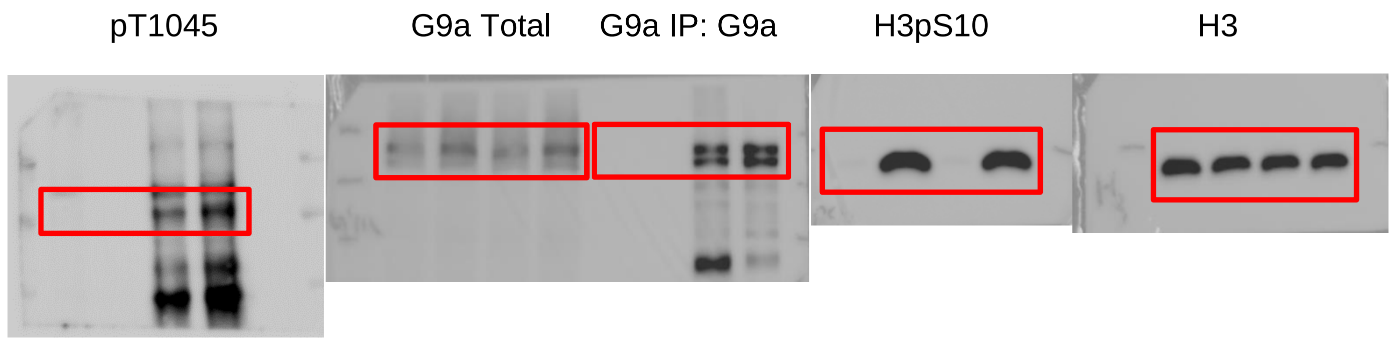

Figure 3

Fig. 3B

H3K9me2 H3 pT1045 Plk1 GST

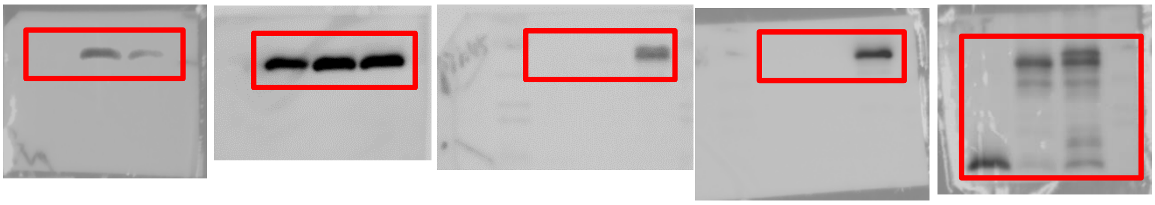

Fig.3 C

GST-G9a-SET	+	+	+	+	+	-
GST	-	-	-	-	+	-
GST-Plk1-KD	+	+	+	+	-	+
H3	+	+	-	-	+	+
ATP	+	-	+	-	+	+
Exp 1	174536	218655	3838	5585	229551	4250
Exp 2	146436	249909	3362	13054	229842	1895
Exp 3	255263	317076	5070	11928	263271	6518

Fig. 3E

H3K9me2 H3 pT1045-G9a Plk1

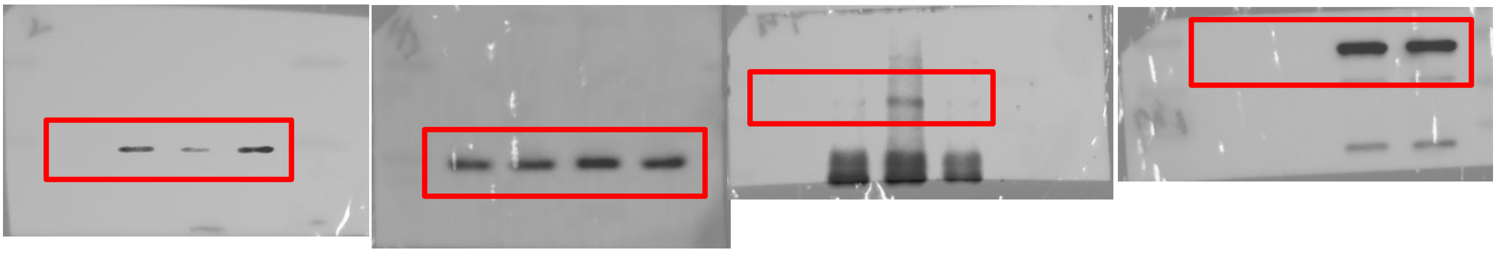

Flag IP: Flag

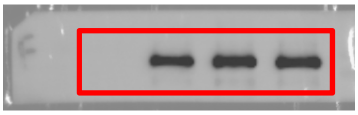

Flag Total

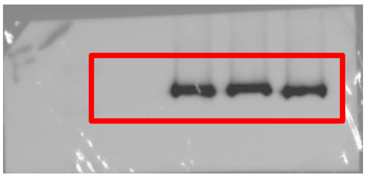

Tubulin

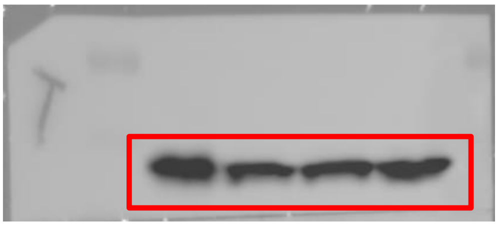

Fig. 3F

rPlk1	-	+	+
BI2536	-	-	+
Exp 1	1	0.859064	1.171715
Exp 2	1	0.591142	1.181358
Exp 3	1	0.528904	1.037575

(Figure 3 continued)

Fig. 3G

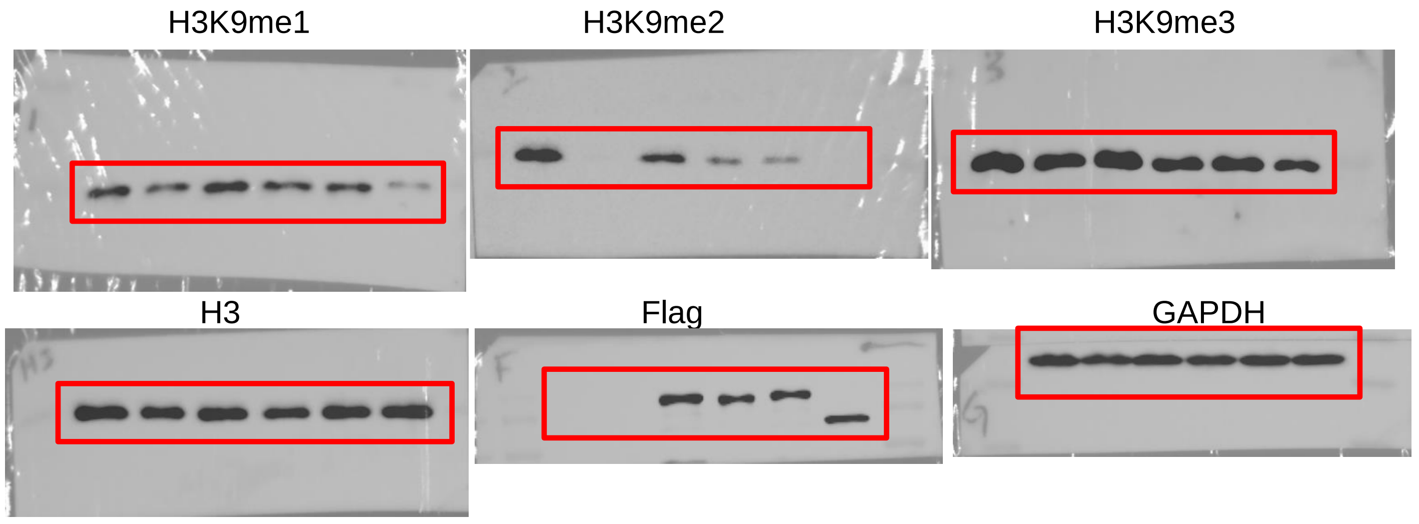

Fig. 3H

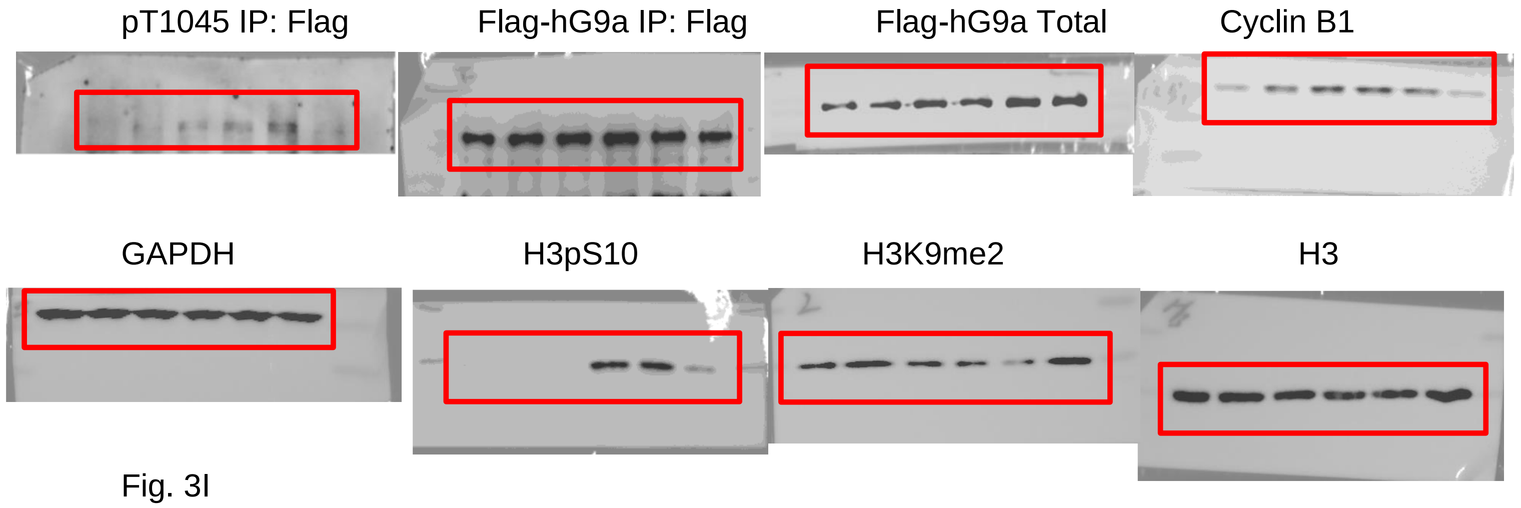

Fig. 3I

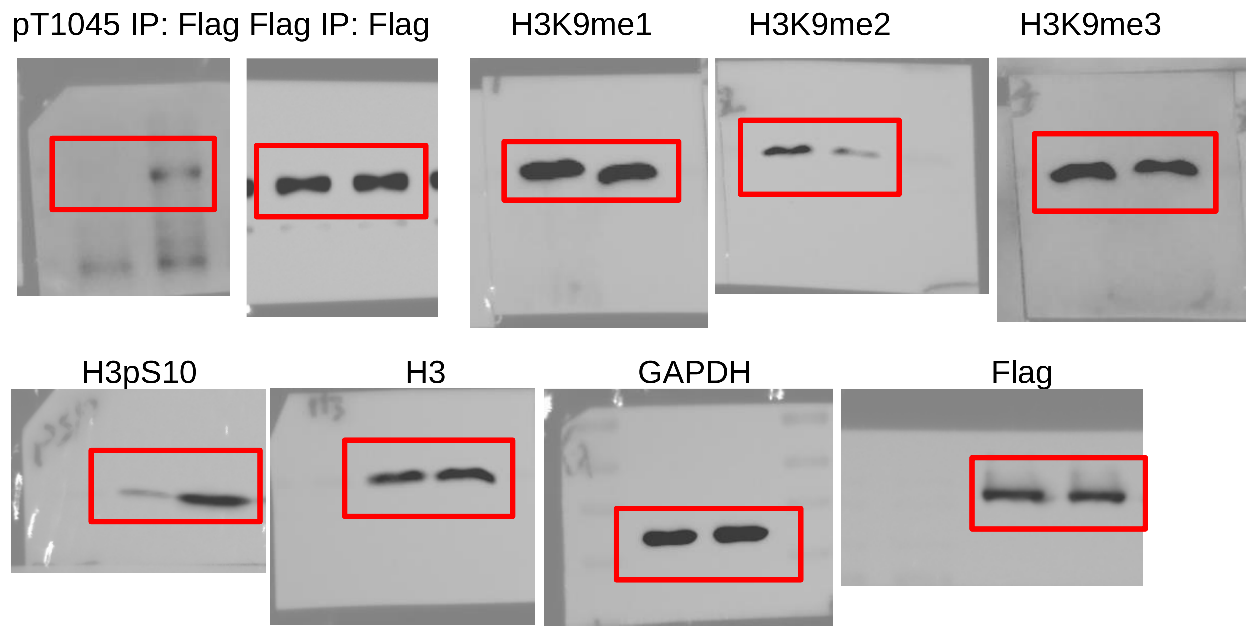

Figure 4

Fig. 4B

	VEC	WT	T1045S	T1045E	DSET	T1045A
Exp 1	1	0.6406488	0.4783583	1.083162	1.302088	0.8701497
	0.9773277	0.6258625	0.4277265	1.068465	1.240255	0.9362846
	0.9320728	0.6674433	0.4295188	1.203692	1.352989	0.9792992
Exp 2	1	0.5584386	0.3737047	0.8871762	0.8650259	0.7853627
	0.9217616	0.595752	0.4034974	0.9190415	0.8137306	0.826943
	0.9091969	0.658783	0.3790155	1.055959	1.017746	0.8613989
Exp 3	1	0.5219337	0.451507	0.9489704	1.180842	0.7866897
	1.08117	0.5443152	0.4428529	1.00746	1.088929	0.7785248
	1.03014	0.5180543	0.4055506	0.9973142	1.036407	0.7889358

Fig. 4C

HA-hG9a GAPDH

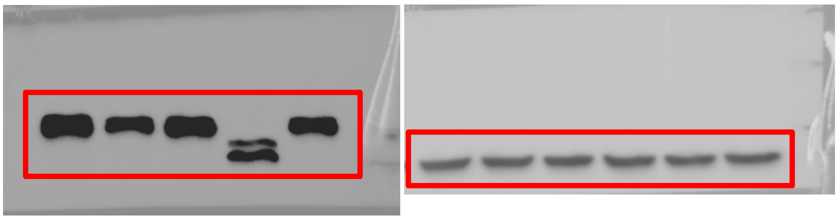

Fig. 4D

Gal4-HA-G9a	Vec			WT		
HA-Plk1	Vec	WT	82M	Vec	WT	82M
Exp 1	1	0.9686661	0.6996713	0.5219337	0.7051626	0.6031036
	1.08117	0.910176	0.7657126	0.5443152	0.6962101	0.5628171
	1.03014	0.9185318	0.8961516	0.5180543	0.6580125	0.5580423
Exp 2	1	1.201697	1.132791	0.5472645	0.5768698	0.5022507
	0.8834834	1.09401	1.034972	0.550554	0.6019737	0.4954986
	0.9319598	1.155125	1.193906	0.5389543	0.6094183	0.518698
Exp 3	1	1.099691	0.9655772	0.471006	0.4914699	0.4467052
	1.180139	1.090795	0.9394701	0.4208994	0.6083194	0.4725615
	1.102785	1.065364	0.748888	0.4194093	0.489747	0.391452

(Figure 4 continued)

Fig. 4E

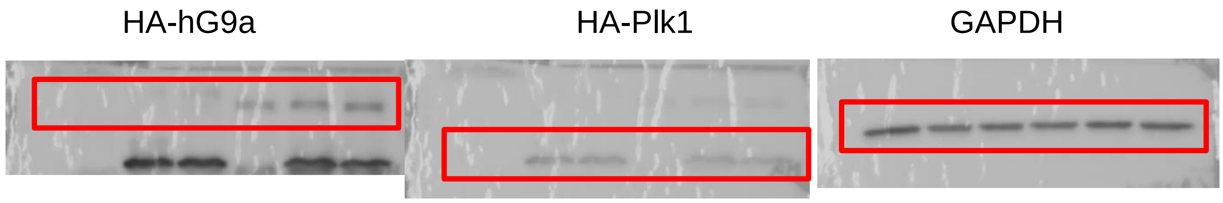

Fig. 4J

	GBA-promoter			
Gal4-HA-G9a	Vec	WT	T1045E	T1045S
Exp 1	1	0.530594	0.928574	0.598323
	1.008242	0.528846	0.854075	0.562937
	1.047203	0.507867	0.81514	0.497502
Exp 2	1.106723	0.576332	0.743546	0.531165
	1	0.550652	0.767425	0.501613
	1.090592	0.575687	0.852215	0.494644
Exp 3	1	0.426524	0.802419	0.440175
	0.973329	0.424047	0.780948	0.405865
	1.073073	0.362099	0.825175	0.326511

	FAM83H-promoter			
Gal4-HA-G9a	Vec	WT	T1045E	T1045S
Exp 1	1	0.533	0.815	0.563
	1.015	0.563	0.978	0.548
	1.119	0.489	0.815	0.6
Exp 2	1	0.534161	0.708075	0.540373
	1.055901	0.478261	0.677019	0.559006
	1.018634	0.509317	0.770186	0.552795

Fig. 4K

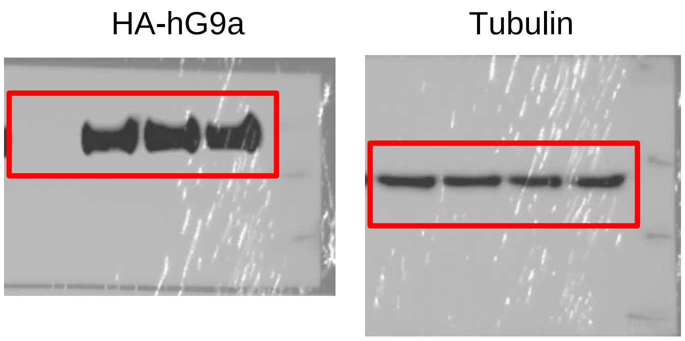

Figure 6

Fig. 6C

	9 h				10 h			
	G1		G2/M		G1		G2/M	
HeLa s3 G9a KO +Flag-hG9a	WT	T1045E	WT	T1045E	WT	T1045E	WT	T1045E
Exp 1	12.8	29.2	77.5	75.3	21.5	19.7	69.8	65
Exp 2	9.23	15.4	82.7	70.9	19.5	18.9	68.4	64.4
Exp 3	10.8	16.6	80	68.7	18.5	21.1	73.9	63.4

	11 h				12 h			
	G1		G2/M		G1		G2/M	
HeLa s3 G9a KO +Flag-hG9a	WT	T1045E	WT	T1045E	WT	T1045E	WT	T1045E
Exp 1	60	34.1	24	53.9	74.9	58.2	14.3	27.6
Exp 2	57.5	35	27.2	49.9	72	57.1	11	23.7
Exp 3	59.6	35.2	29.3	53	72.6	59.8	14.8	26.9

Fig. 6D

	9 h		10 h		11 h		12 h	
	WT	T1045E	WT	T1045E	WT	T1045E	WT	T1045E
Exp1	23.4	22.4	34.3	30.1	21.7	27.8	12.6	20.4
Exp2	25.4	24.7	38.1	29	23.3	27.6	14.6	23.3
Exp3	20.9	18.8	33.3	28.2	21.9	27.7	11.8	20.9

Fig. 6E

	Prometaphase							
	9 h		10 h		11 h		12 h	
HeLa s3 KO +Flag-hG9a	WT	T1045E	WT	T1045E	WT	T1045E	WT	T1045E
Exp 1	0	4.76	18.18	8	4.35	5.56	4.76	0
	0	0	15.38	0	11.11	12	4	5.56
	0	0	7.14	12	0	16.67	8.33	5.56

	0	0	5.26	16	8.33	8.33	0	0
	4.35	0	17.65	0	5	12.5	3.45	4.55
Exp 2	9.09	0	11.11	5.26	0	3.125	0	0
	0	0	18.75	3.03	4.167	7.143	3.23	4.88
	0	3.45	7.14	7.14	4.167	10	2.94	4.35
	0	0	12	5	6.25	9.091	2.78	2.94
	0	5.88	13.79	4.55	0	6.667	3.33	6.9
Exp 3	0	0	13.33	17.65	0	0	3.57	6.25
	0	0	11.54	13.04	16.67	5.26	5	3.33
	0	4.17	8.33	11.11	0	0	3.13	0
	0	0	18.18	8.33	5.26	0	3.85	3.23
	0	0	8.33	6.45	0	3.23	0	0

	Metaphase							
	9 h		10 h		11 h		12 h	
HeLa s3 KO +Flag-hG9a	WT	T1045E	WT	T1045E	WT	T1045E	WT	T1045E
Exp 1	0	4.76	36.36	8	17.39	22.22	0	5.88
	5	0	23.08	5.56	0	36	8	11.11
	4.76	0	7.14	12	0	25	0	0
	0	0	5.26	4	0	20.83	0	0
	0	0	17.65	5	15	25	3.45	13.64
Exp 2	0	3.13	25.93	18.42	4	12.5	0	3.7
	3.7	0	12.5	12.12	0	16.67	6.45	4.88
	0	0	28.57	14.29	4.17	12.5	0	8.7
	0	3.7	28	10	12.5	20.45	0	8.82
	0	2.94	20.69	13.64	5.26	17.78	0	10.34
Exp 3	0	4.55	6.67	11.76	0	4	0	3.13
	0	0	11.54	8.7	0	5.26	0	3.33
	0	4.17	8.33	11.11	5.56	0	3.13	5.26
	0	0	9.09	8.33	5.26	0	3.85	9.68
	0	0	8.33	9.68	15.79	3.23	0	0

	Anaphase							
	9 h		10 h		11 h		12 h	
HeLa s3 KO +Flag-hG9a	WT	T1045E	WT	T1045E	WT	T1045E	WT	T1045E
Exp 1	0	0	13.64	8	0	22.22	9.52	5.88
	5	5	7.69	0	0	4	4	5.56
	0	0	7.14	4	0	8.33	8.33	5.56
	0	0	15.79	4	4.17	12.5	0	0
	4.35	0	5.88	0	5	4.17	0	4.55
Exp 2	0	0	22.22	5.26	4	6.25	4.55	3.7
	0	0	25	0	8.33	4.76	3.23	7.32
	0	0	14.29	7.14	12.5	7.5	5.88	13.04
	0	0	12	5	12.5	11.36	5.56	5.88
	0	0	13.79	2.27	10.53	6.67	10	3.45
	0	0	0	11.76	0	12	7.14	6.25
	0	0	7.69	0	0	10.53	5	3.33
	0	4.17	0	3.7	0	7.14	3.13	5.26
	0	0	18.18	8.33	10.53	8.7	11.54	3.23
	0	0	0	6.45	5.26	3.23	0	0

	Telophase							
	9 h		10 h		11 h		12 h	
HeLa s3 KO +Flag-hG9a	WT	T1045E	WT	T1045E	WT	T1045E	WT	T1045E
Exp 1	0	0	9.09	0	17.39	5.56	52.38	23.53
	0	5	23.08	0	33.33	4	52	44.44
	4.76	0	0	4	27.27	8.33	66.67	38.89
	0	0	0	12	33.33	8.33	61.11	45
	0	0	5.88	5	20	4.17	48.28	13.64
Exp 2	0	0	3.7	0	20	6.25	63.64	48.15
	0	0	0	0	58.33	14.29	51.61	39.02
	0	0	3.57	4.76	29.17	10	50	43.48
	0	0	12	2.5	31.25	4.55	50	47.06
	0	0	6.9	4.55	47.37	4.44	43.33	27.59

Exp 3	0	0	0	0	52.63	4	57.14	40.63
	0	0	3.85	0	33.33	42.11	60	43.33
	4.76	0	0	0	16.67	35.71	43.75	57.89
	0	0	0	0	21.05	30.43	57.69	38.71
	0	0	0	0	10.53	35.48	77.27	42.31

Fig. 6F

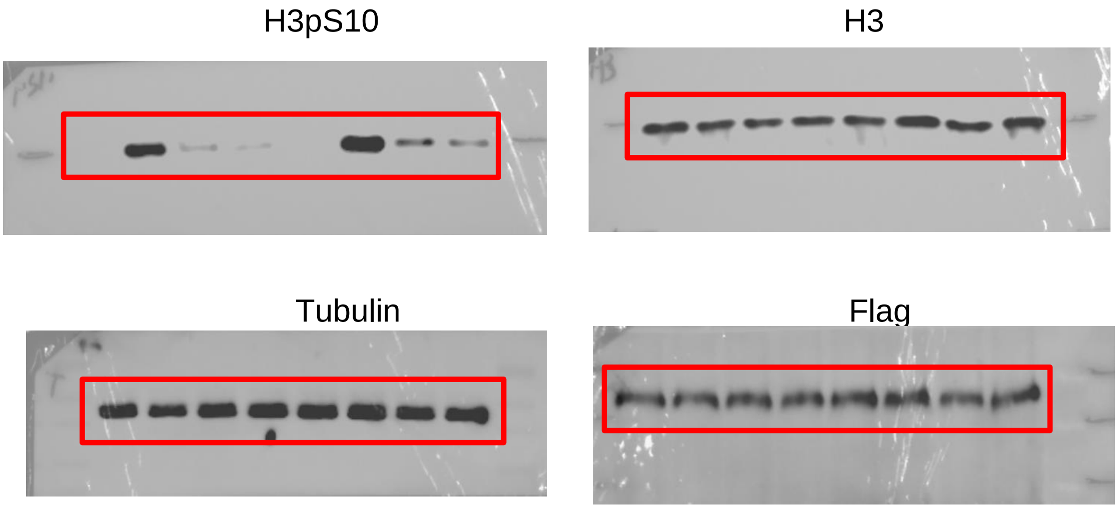

Fig. 6G

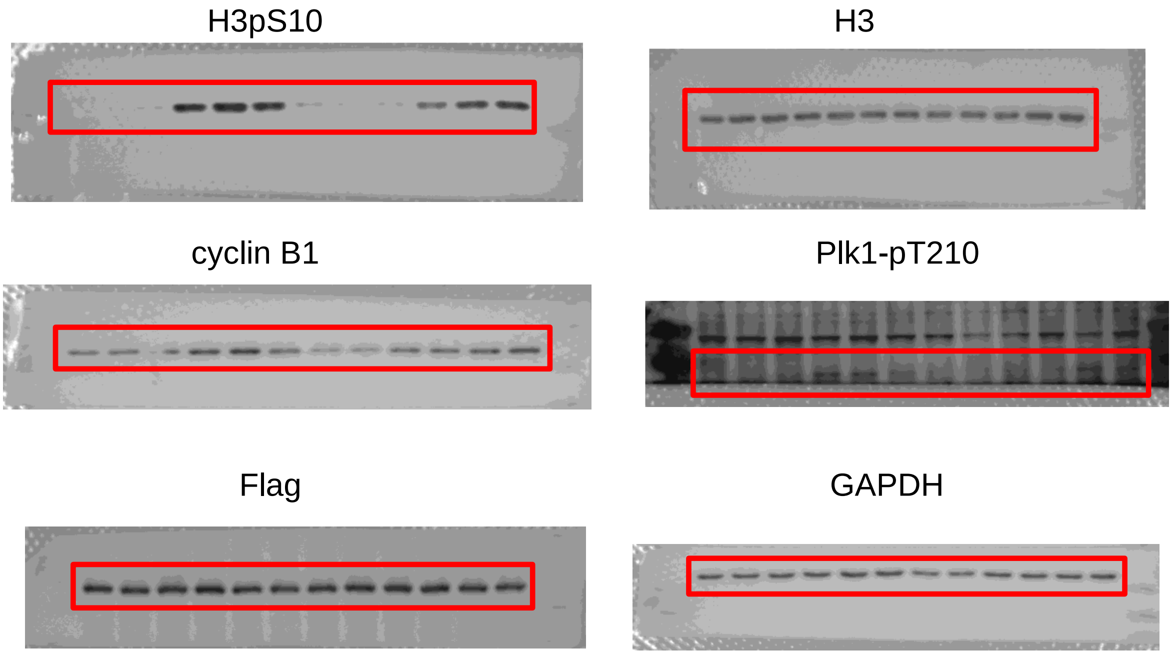

Figure 7

Fig. 7A

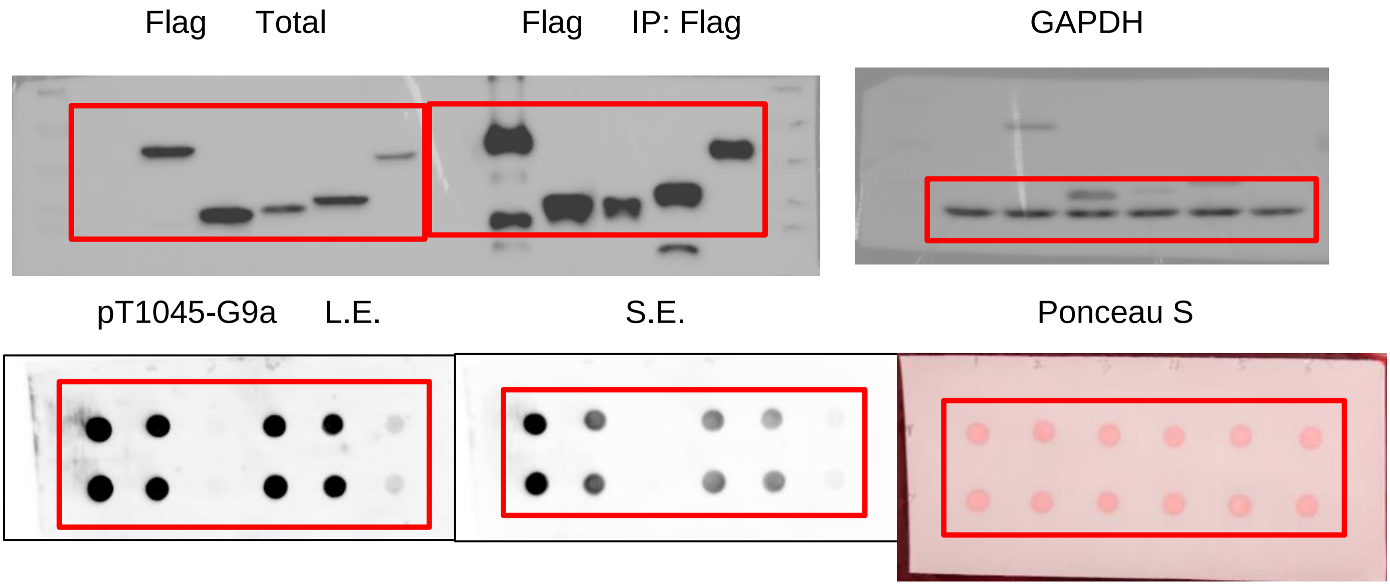

Fig. 7B

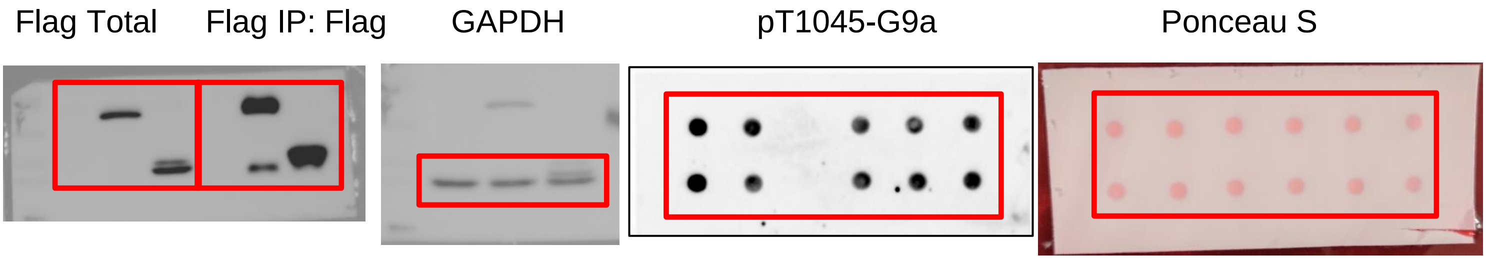

Fig. 7C

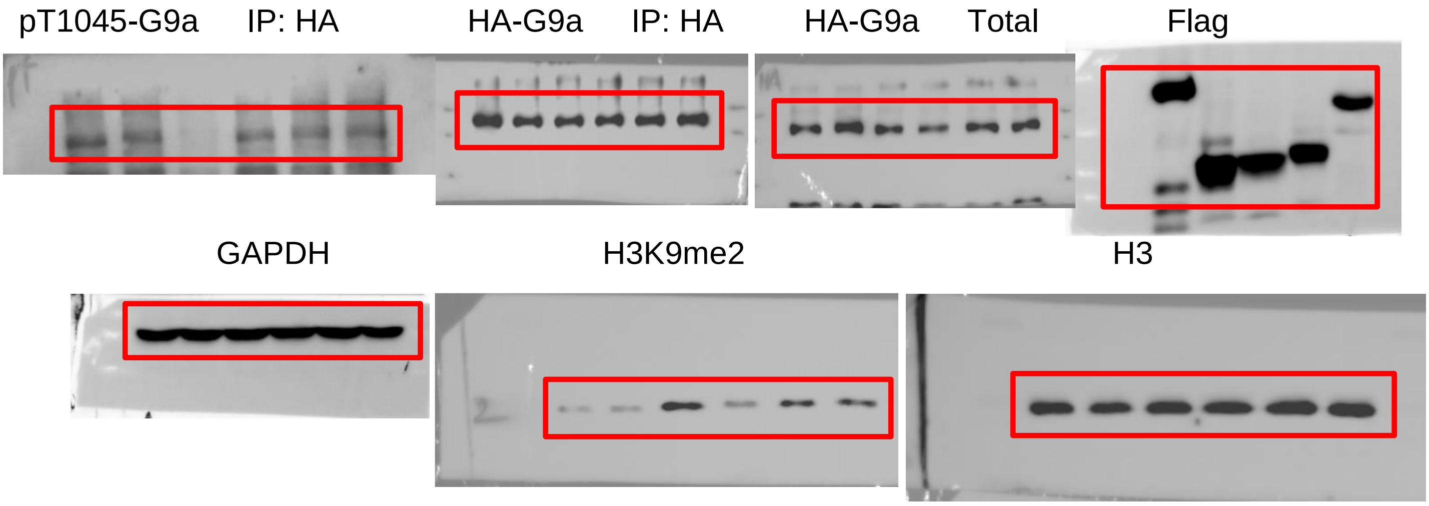

(Figure 7 continued)

Fig. 7D

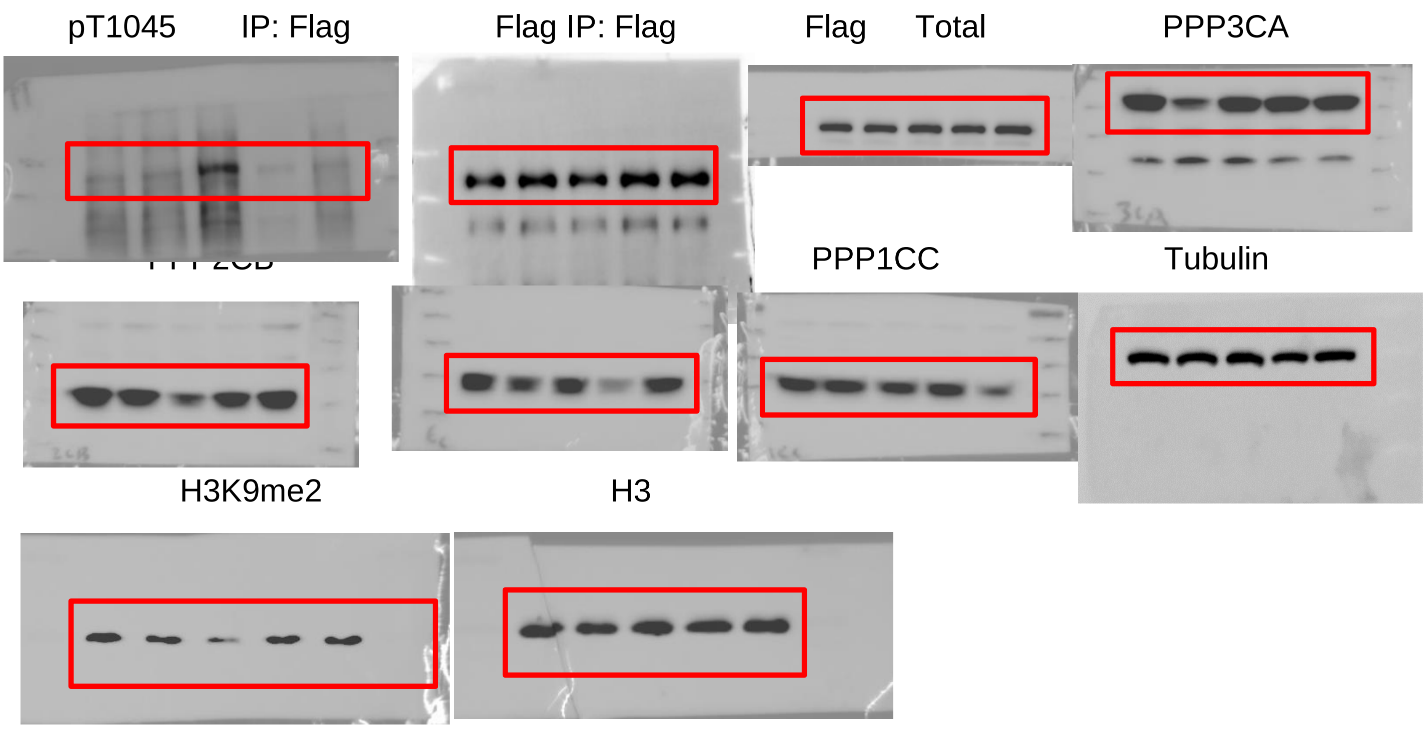

Fig. 7E

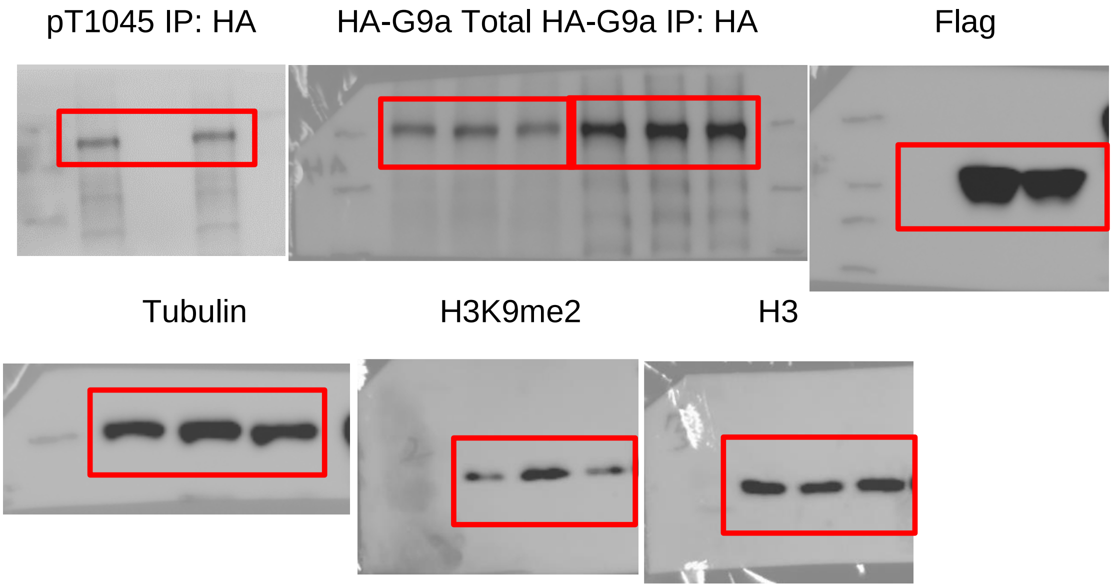

Fig. 7F

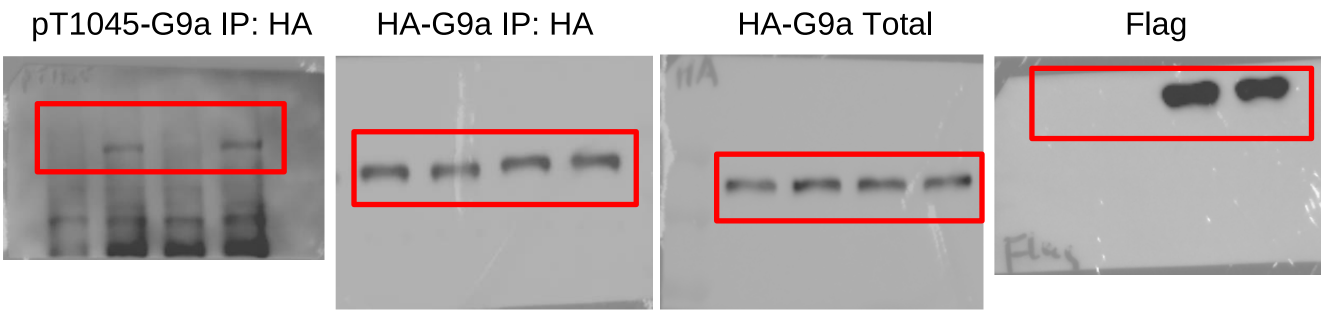

PPP2CB

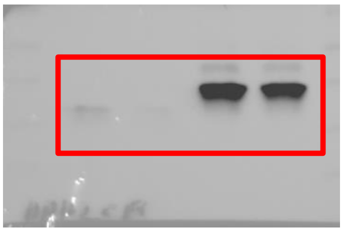

Tubulin

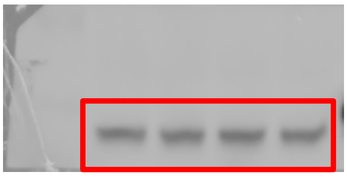

H3K9me2

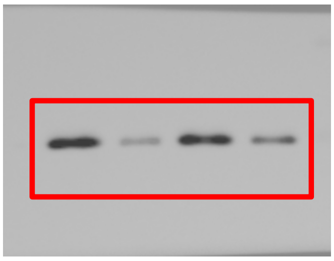

H3

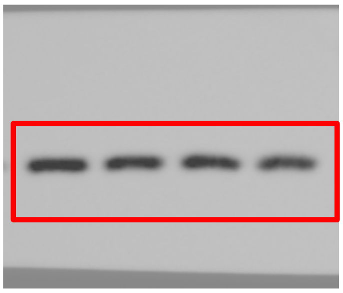

Fig. 7G

pY307-PPP2CB

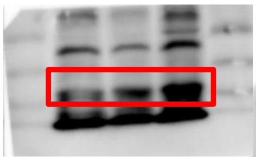

PPP2CB

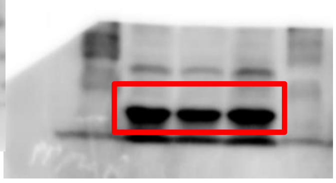

GAPDH

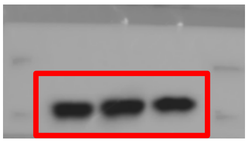

H3pS10 H3

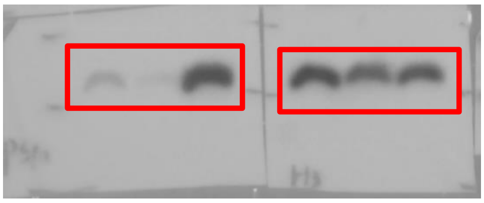

Fig.7H

pT1045 IP: Flag

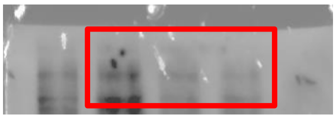

Flag IP: Flag

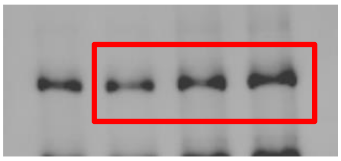

H3pS10

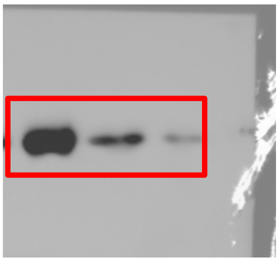

H3K9me2

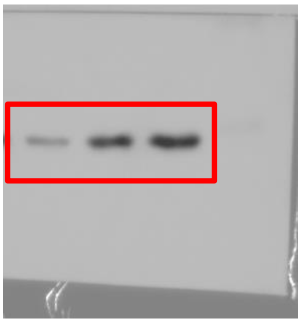

pY307

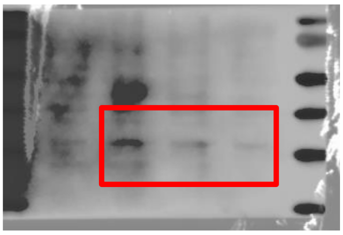

PPP2CB

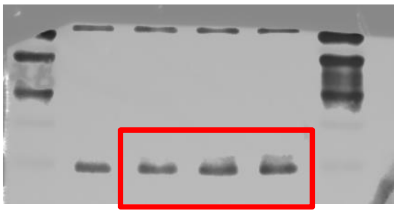

Flag-G9a Total

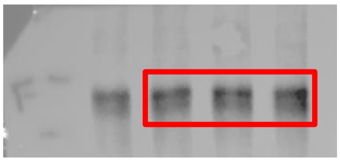

H3

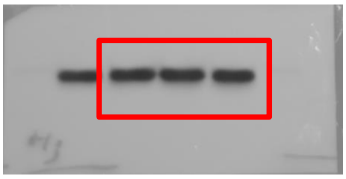

Tubulin

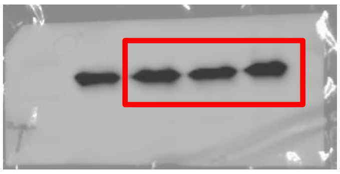

Figure S1

Figure S1E

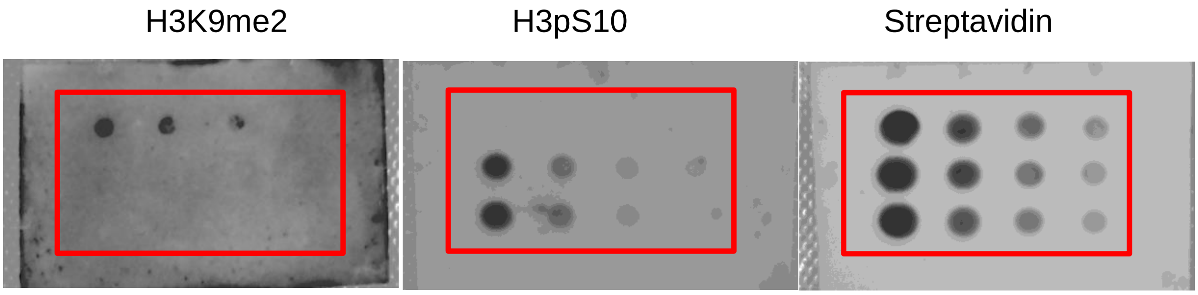

Figure S3

Fig. S3A

Ponceau pT1045

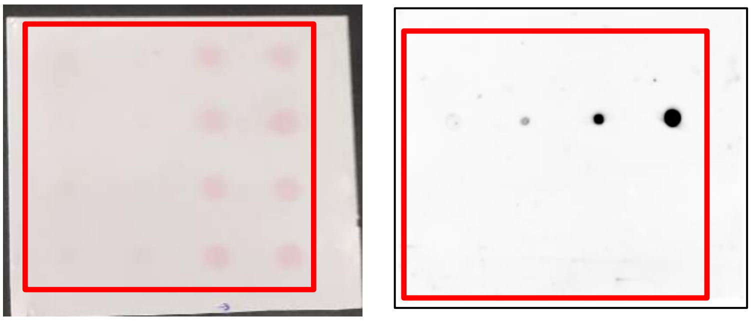

Fig. S3B

Ponceau pT1045

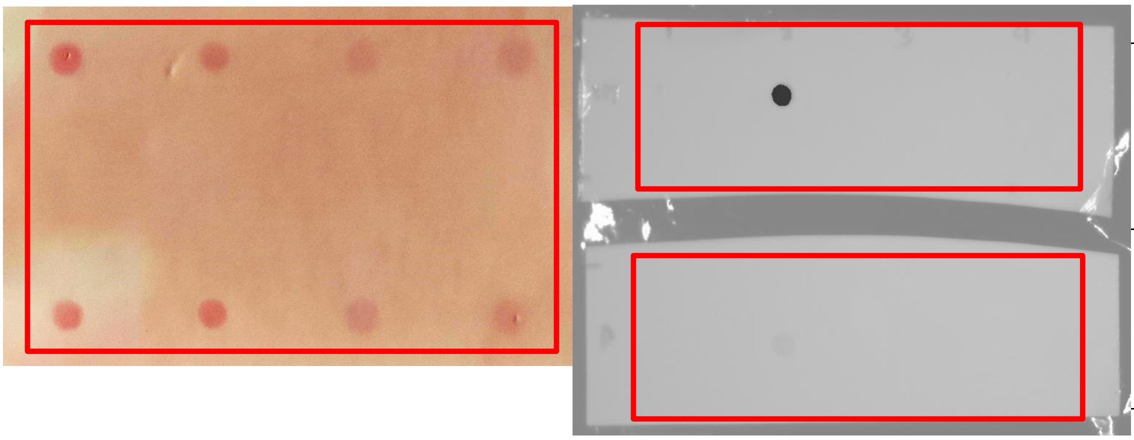

antibody +WT peptide

antibody +pT1045 peptide

Fig. S3C

Flag

pT1045 IP: Flag Total IP: Flag actin

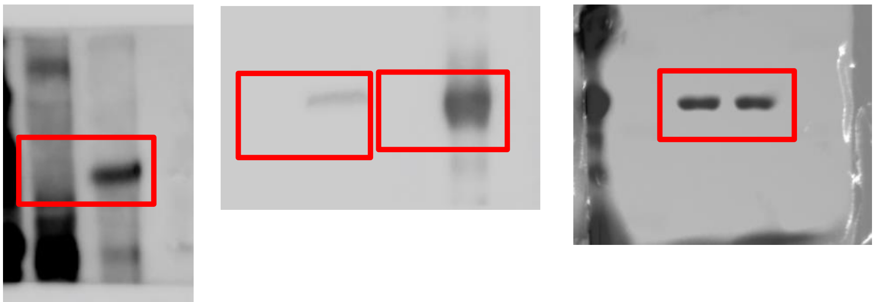

Figure S4

Fig. S4A

pT1045 Plk1 GST

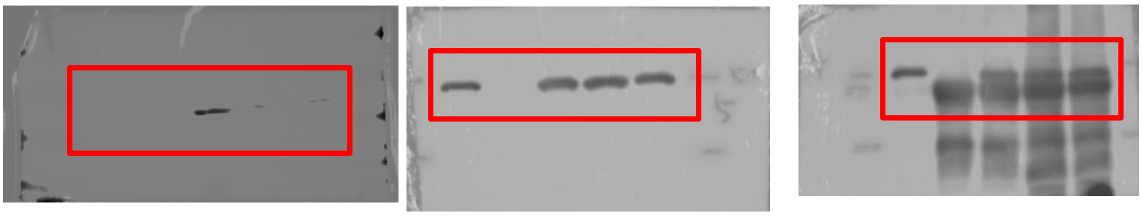

Fig. S4B

Flag

pT1045 IP: Flag Total IP: Flag HA-Plk1 actin

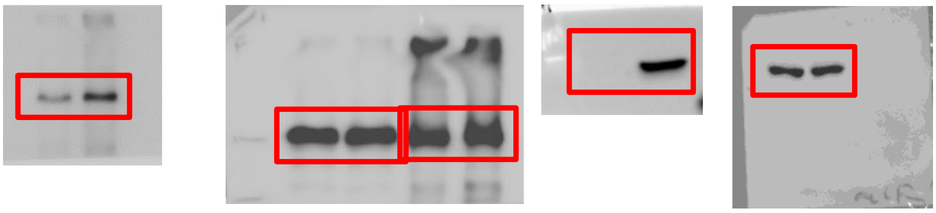

Fig. S4C

Flag

pT1045 IP: Flag IP: Flag Total actin

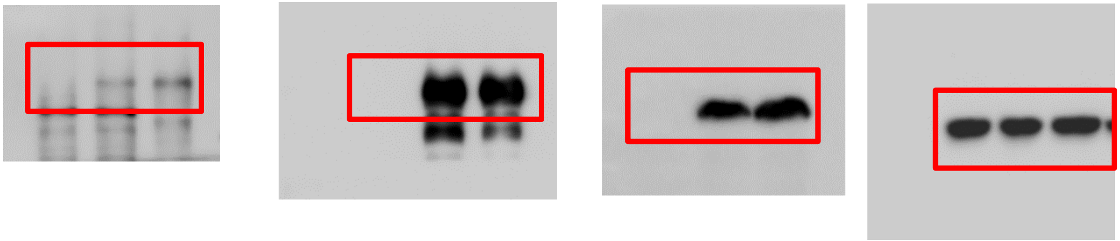

Figure S5

Fig. S5B

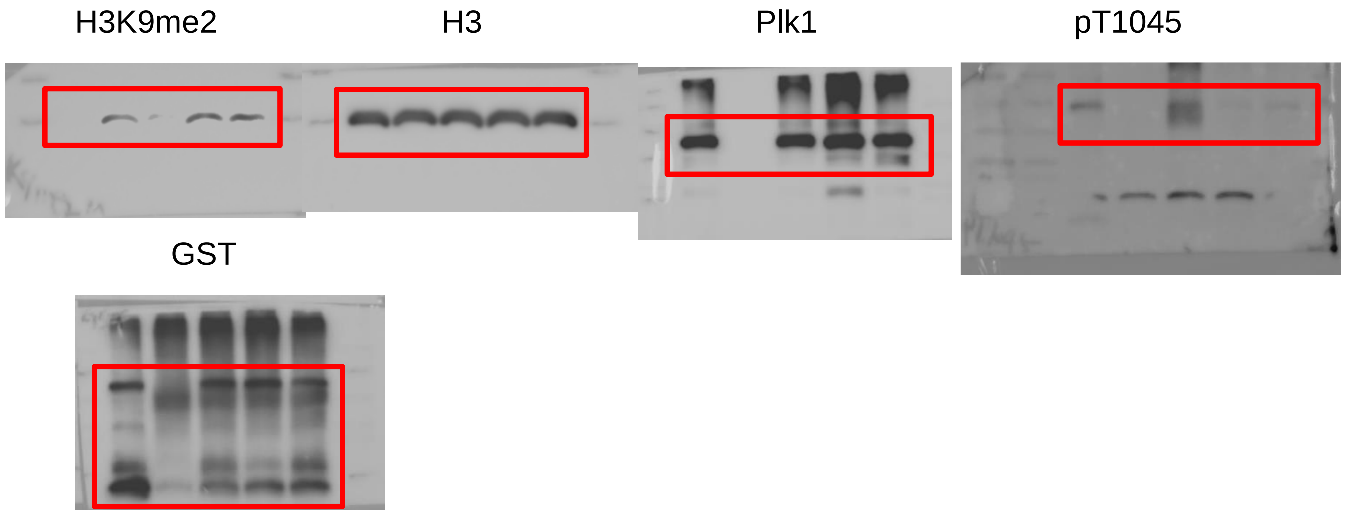

Fig. S5C

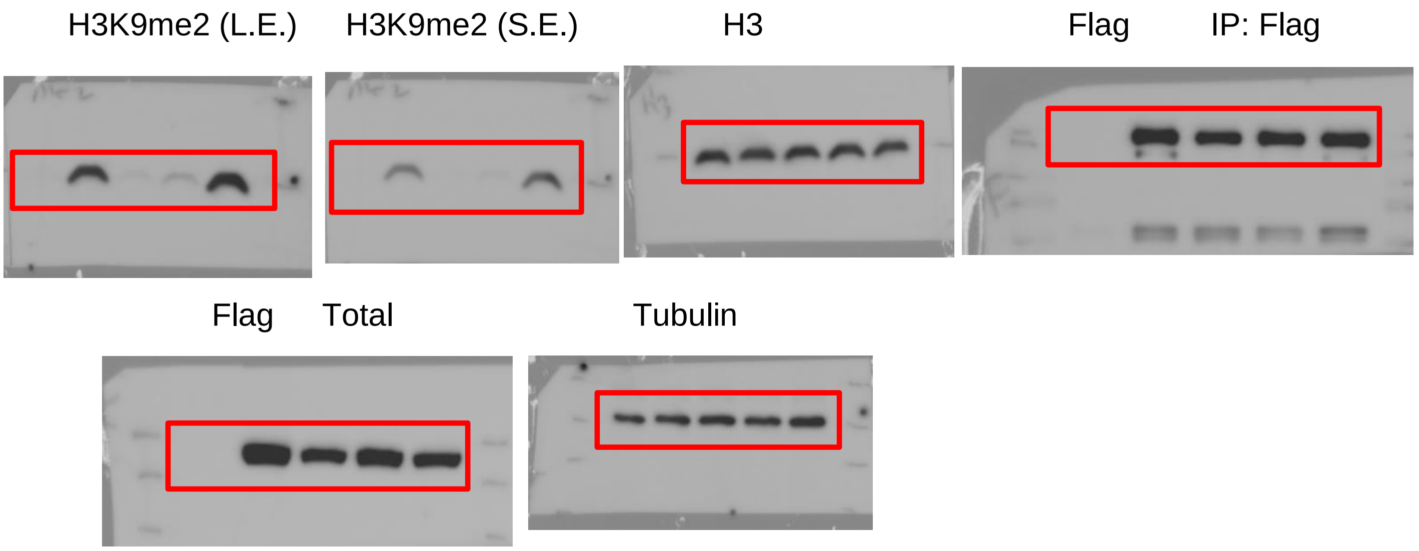

Fig. S5D

Flag-hG9a	vec	WT	T1045E	T1045A	T1045S
Exp 1	292	3296	1085	1572	2615
Exp 2	525	3849	2260	2401	3981
Exp 3	428	5815	1343	1746	6045

Figure S7

Fig. S7A

	WT Plk1	K82M Plk1	WT Plk1	K82M Plk1	WT Plk1	K82M Plk1	WT Plk1	K82M Plk1
	WT	WT	T1045E	T1045E	T1045A	T1045A	T1045S	T1045S
Exp1-1	1.1964	0.6988	1.2751	1.6133	1.286	1.5042	0.9792	0.8537
Exp1-2	1	0.6982	1.3956	1.6215	1.4059	1.424	0.9235	0.7073
Exp1-3	1.1108	0.6458	1.2579	1.7025	1.255	1.5126	0.8936	0.8943
Exp2-1	1.045	0.781	1.626	1.249	1.332	1.01	0.7312	1.061
Exp2-2	1.138	0.889	1.948	1.236	1.413	1.041	0.8674	1.088
Exp2-3	1	0.839	1.644	1.21	1.282	1.149	0.9612	0.946

Fig. S7B

HA-G9a HA-Plk1 GAPDH

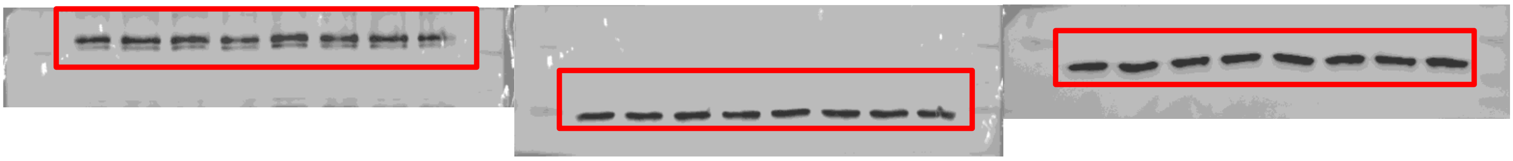

Fig. S7C

Flag-hG9a GAPDH

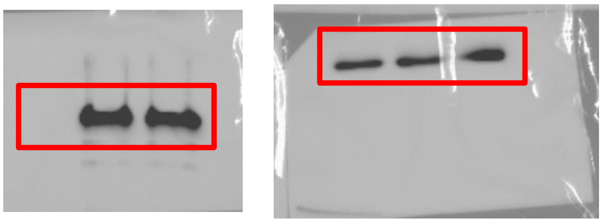

Fig. S6F

HA-G9a Tubulin

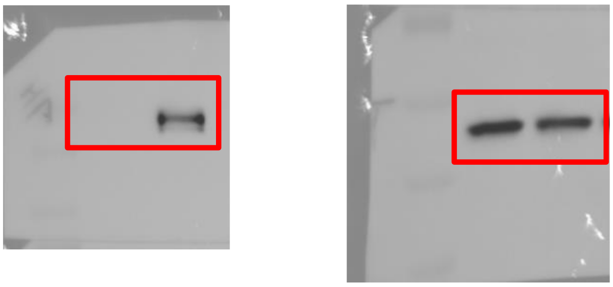

Figure S8

Fig. S8A

G9a Flag-hG9a Tubulin

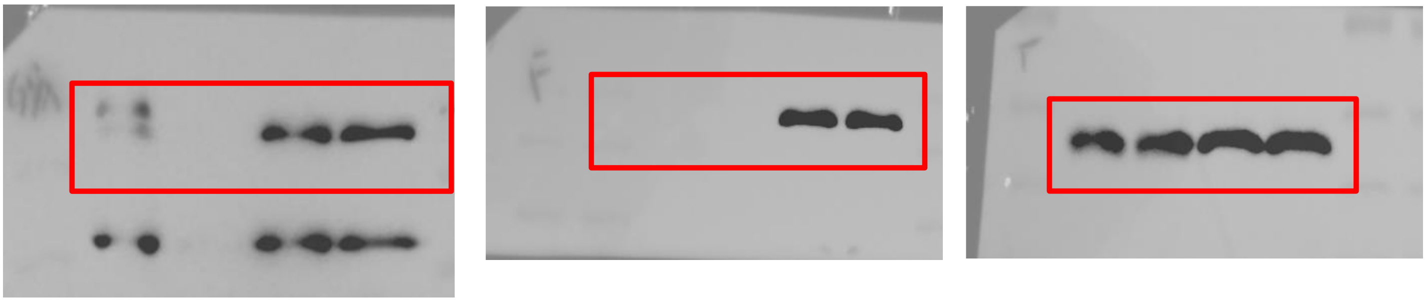

Figure S9

Fig. S9A

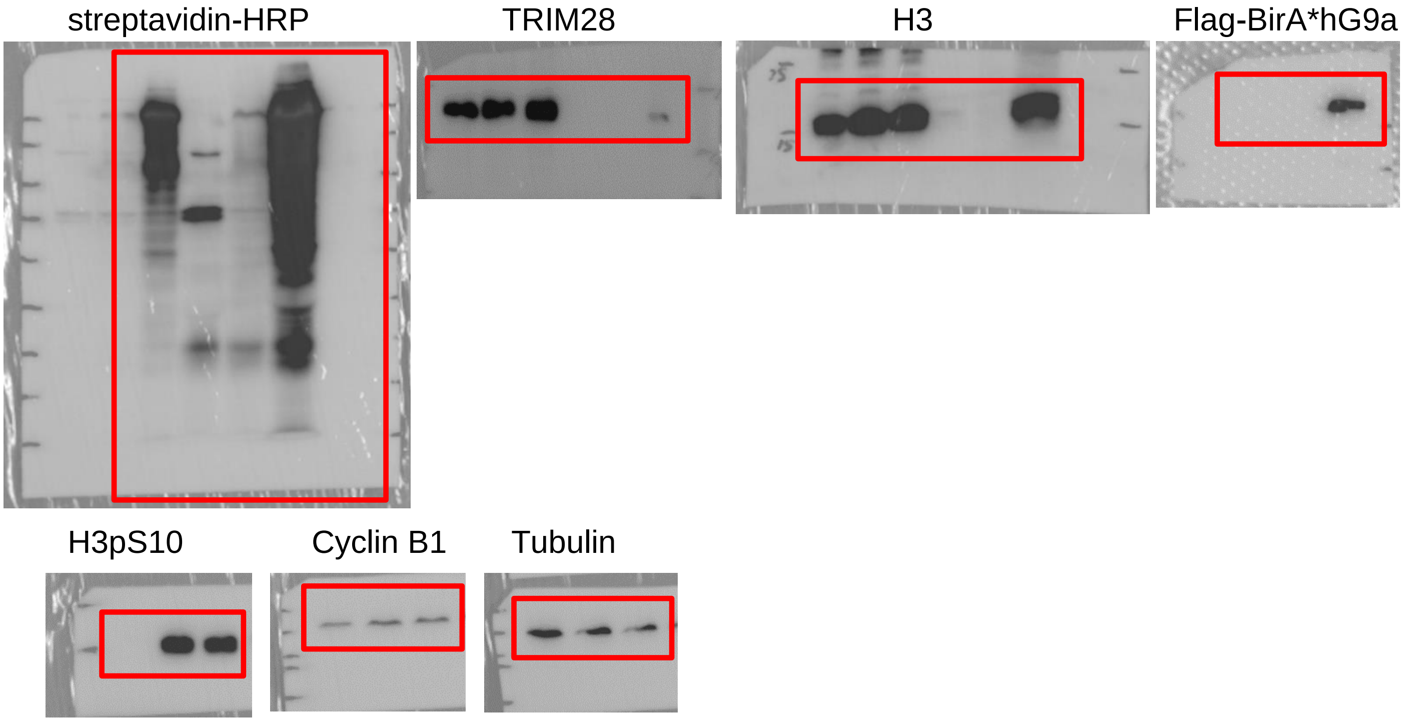

Supplement: Supplementary file 2 — Supporting Information 2 [file ADVS-10-2303224-s001.pdf]
